# New insights into the *Plasmodium vivax* transcriptome using RNA-Seq

**DOI:** 10.1038/srep20498

**Published:** 2016-02-09

**Authors:** Lei Zhu, Sachel Mok, Mallika Imwong, Anchalee Jaidee, Bruce Russell, Francois Nosten, Nicholas P. Day, Nicholas J. White, Peter R. Preiser, Zbynek Bozdech

**Affiliations:** 1 School of Biological Sciences, Nanyang Technological University, Singapore; 2 Mahidol-Oxford Tropical Medicine Research Unit, Faculty of Tropical Medicine, Mahidol University, Bangkok, Thailand; 3 Centre for Tropical Medicine and Global Health, Nuffield Department of Medicine, University of Oxford, Oxford, UK; 4 Shoklo Malaria Research Unit, Mahidol-Oxford Tropical Medicine Research Unit, Faculty of Tropical Medicine, Mahidol University, Mae Sot, Thailand; 5 Yong Loo Lin School of Medicine, National University Singapore, Singapore

## Abstract

Historically seen as a benign disease, it is now becoming clear that *Plasmodium vivax* can cause significant morbidity. Effective control strategies targeting *P. vivax* malaria is hindered by our limited understanding of *vivax* biology. Here we established the *P. vivax* transcriptome of the Intraerythrocytic Developmental Cycle (IDC) of two clinical isolates in high resolution by Illumina HiSeq platform. The detailed map of transcriptome generates new insights into regulatory mechanisms of individual genes and reveals their intimate relationship with specific biological functions. A transcriptional hotspot of *vir* genes observed on chromosome 2 suggests a potential active site modulating immune evasion of the *Plasmodium* parasite across patients. Compared to other eukaryotes, *P. vivax* genes tend to have unusually long 5′ untranslated regions and also present multiple transcription start sites. In contrast, alternative splicing is rare in *P. vivax* but its association with the late schizont stage suggests some of its significance for gene function. The newly identified transcripts, including up to 179 *vir* like genes and 3018 noncoding RNAs suggest an important role of these gene/transcript classes in strain specific transcriptional regulation.

Malaria remains a global problem impacting hundreds of million lives around the world. Over recent years significant progress has been made in reducing the global burden of *Plasmodium falciparum* and major steps towards an operational vaccine have been recently made^1^,^2^. The successful control of *P. falciparum* has though highlighted how little progress has been made to control the second species of the malaria pathogen; the geographically most widely spread parasite, *P. vivax*. It is of particular concern that in areas where *P. falciparum* control is successful, *P. vivax* becomes dominant^3^. This suggests that the overall morbidity caused by *P. vivax* has been significantly underestimated in the past^4^,^5^,^6^. The distinct pathophysiology of *P. vivax* that is now being fully appreciated, calls for specific disease control programs that need to be different from those that until now are being used in most endemic regions to target *P. falciparum*^3^,^5^,^7^.

The specificities of *vivax* malaria are underlined by unique biology of the pathogen itself and its interaction with the host^7^,^8^,^9^. The difficulty in studying this parasite in the laboratory due to a lack of a continuous *in vitro* culture system, limits the understanding of molecular mechanisms that characterize this parasite. Nonetheless publication of the genome^10^ and the transcriptome of the blood stage life cycle^11^,^12^ in 2008 provided invaluable insights into *P. vivax* biology. The subsequent high throughput sequencing^13^ and the assembly of a genetic map helped us to understand the genetic diversity of this pathogen^9^,^14^,^15^,^16^,^17^,^18^ showing specific differences from other parasites^10^,^19^,^20^,^21^. Altogether these genomic studies provided a broader platform for identifying new molecular targets for malaria intervention strategies^22^,^23^,^24^,^25^. More insights were also obtained into the genetic nature of *P. vivax* relapses resulting from the activation of the dormant hypnozoite stage^26^.

Genome-wide transcriptional analyses throughout the life cycle stages of *Plasmodium* using microarray approaches revealed many features of gene expression and utilization in respect to both the parasite growth and adaptation to its host^10^,^11^,^12^. Nonetheless, some properties of gene expression remained uncharacterized including absolute expression levels, the structures of the untranslated regions (UTRs) or the full extent of alternative splicing (AltSpl). RNA-Seq studies of *P. falciparum* parasites failed in deriving these information due to the high abundance of AT-rich low complexity sequences in the genome^27^,^28^,^29^. Although the *P. vivax* genome is highly syntenic to *P. falciparum* with only a handful of syntenic breaks within the 14 chromosomes, it has a higher CG content (45% CG) and virtually lacks the A-T rich repeats^10^,^30^. Here we establish the *P. vivax* transcriptome of the Intraerythrocytic Developmental Cycle (IDC) of two clinical isolates in high resolution by Illumina HiSeq platform. This study complements the previous microarray study^11^ providing a better understanding of the dynamics of the *P. vivax* transcriptome by measuring the absolute transcript levels. In addition, we complete and expand the *P. vivax* gene annotation by characterizing UTRs and transcriptional start sites (TSS) for the majority of the genes, identifying new AltSpl events and also previously unknown protein-coding and noncoding transcripts.

## Results and Discussion

### Sequencing of the *P. vivax* transcriptome

The global transcriptome of human malaria parasites has been previously characterized by microarray-based studies showing that the vast majority of genes display a single peak abundance profile that correlate with their functional needs during the IDC of both *P. falciparum*^31^,^32^ and *P. vivax*^11^,^12^. To interrogate the global pattern of *P. vivax* transcription in higher resolution, we applied massively parallel sequencing to the identical RNA samples previously used in the microarray study^11^. Briefly, the *P. vivax* samples were collected from two patients at the Shoklo Malaria Research Unit, Mahidol University, Mae Sot, Northwest Thailand (here defined as SMRU1 and SMRU2). Samples were highly synchronous containing 100% and 83% ring stages at the time of blood collection as validated by microscopy-based parasite morphology counts. High levels of synchrony were subsequently maintained over the 48 hour *ex vivo* cultures with morphological stage exclusivity reaching 100–94% for trophozoites and 82–65% for schizonts at the given time points (for details of sample collection and *ex vivo* culturing see reference^11^). Overall, 15 parasite samples (7 for SMRU1 and 8 from SMRU2 isolates) were used for sequencing (Methods). In addition, four samples that consisted of equal amounts of total RNA from the 15 time course samples were also prepared. These samples provided an important reference for the quality of the sequencing process, statistical evaluation of RNA abundance, and also discovery of UTRs, AltSpl and new transcripts (see below). In the further text, we refer to them as the control reference samples. Illumina HiSeq2000 was employed in a single flow cell to generate a total of 1.7 billion paired-end 2 × 101 bp reads with a median of 81M read pairs per sample (range, 61 M–134 M). There were 3.7% to 12.2% (median = 5.7%) reads uniquely mapping to the *P. vivax* SalI genome^10^,^33^ (PlasmoDB^30^,^34^,^35^ release12) excluding regions of r(t)RNAs and 0.8% to 14.4% reads (median = 2.2%) mapping to human RNAs (see Supplementary Fig. S1 and Table S1 online). There were no reads that mapped to the core genome of other *Plasmodium* species which further confirms that the two donor patients were not infected by other (than *P. vivax*) *Plasmodium* parasites. Overall, 2.2 M to 5.1 M (median = 3.1 M) read pairs per library mapped to the 22.6 M chromosome sequences that resulted in 20–45 fold (median = 27) coverage of the *P. vivax* genome. For the 13,626 exons in the total 5586 annotated protein coding genes in *P. vivax*, 93% of them showed coverage greater than 10-fold in at least one time point across the IDC in both isolates and 88% of the exons showed the same high level of coverage (>10 fold) in the four control reference samples. Reproducibility of the results from the control reference samples was demonstrated by Pearson Correlation Coefficient (PCC) that was greater than 0.99 for each pair which indicates a high fidelity of our sequencing procedures (see Supplementary Fig. S2a–f online).

### mRNA abundance profiles across the *P. vivax* IDC

**Figure 1:**
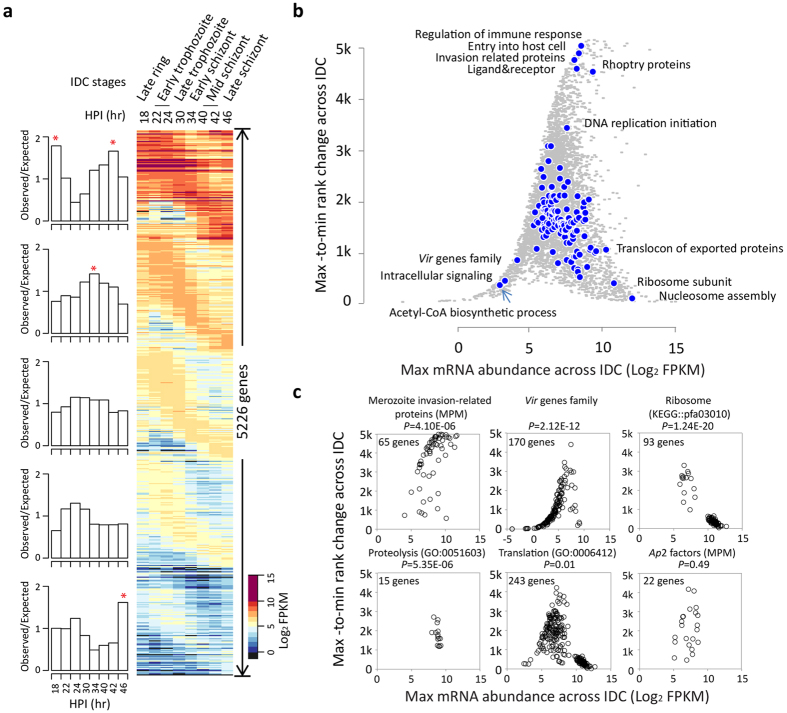
The *P. vivax* IDC transcriptome and its functional aspects. (**a**) The heat map shows the overview of the IDC transcriptome of the SMRU2 *P. vivax* isolate (see Supplementary Fig. S3 online for the SMRU1 transcriptome). Briefly, mRNA abundance (log_2_FPKM) of 5226 annotated protein-coding genes is depicted across the IDC in five groups stratified (20^th^ percentile) based on their maximum level of transcription. Genes were sorted by their expression timing within group. The estimation of timing and parasites age of hours post invasion (HPI) are described in Methods. Left bar plots represent the fold enrichment of time-point specific genes (genes peaking their transcription at the same particular time point during the IDC) for each group. The expected frequency used here is the proportion of genes maximally expressed at the time point in whole genome; * indicates over-representation by binomial test at *P* < 0.01. (**b**) The “wizard’s hat”-like distributions was generated by plotting the transcriptional dynamics (max-to-min rank change of mRNA abundance through the IDC) against the maximum mRNA abundance for 5226 genes (grey dots). Blue dots represent medoids of functional groups (see Supplementary Data S2 online) with member genes significantly (*P* < 0.01) clustered by locations on “wizard’s hat” compared to random dataset. (**c**) Scatter plots for selected pathways/functional groups which are significantly clustered on the top (merozoite proteins), left bottom (*vir* family), right bottom (ribosome proteins) and middle (proteolysis and translation) of the distribution depicted in panel (**b**). In addition, the scatted of the *Ap2* family members is shown.

To reconstruct the transcriptional cascade of the *P. vivax* IDC, we used *Fragments Per Kilobase of coding exon per Million fragments mapped* (FPKM)^36^ to measure the absolute mRNA abundance for each gene in the individual time points. In total, we established expression profiles for 5226 protein coding genes (see Fig. 1a and Supplementary Data S1 online). Single peak transcription profiles were observed for 4981 (95%) genes in the SMRU1 and 4828 (92%) in the SMRU2 time courses, respectively. These RNA-Seq-derived transcriptional profiles agreed with the microarray-based results^11^ with correlations values 0.95 ± 0.06 (median PCC ± Median absolute deviation) and 0.91 ± 0.1 for SMRU1 and SMRU2 isolates, respectively (see Supplementary Fig. S4a,b online). Concordant with our previous study, the expression timing is highly conserved between the isolates with a median PCC of 0.85; only 43 genes exhibit significant difference in timing of its transcription (see Supplementary Table S2 online). Finally, the dynamic range (max-to-min change) of mRNA abundance were improved approximately 3-fold by RNA-Seq compared to the microarrays (see Supplementary Fig. S4c online).

Our results also correlated well with two microarray based *P. vivax* transcriptomic studies: (i) 3918 (75 %) of the genes identified here were also found to change their overall expression by more than two fold across the IDC by Westenberger *et al.*^12^; (ii) 4351 genes (83%) out of total 5226 genes found by the RNA-Seq approach was also found amongst the sense strand transcripts by Boopathi *et al.*^37^ (see Supplementary Fig. S4d,e online).

### Functional relevance of the *P. vivax* IDC transcriptional program

To investigate the biological significance of the absolute expression levels, we stratified the 5226 detected genes into five groups (20th percentiles each group) based on their peak levels of mRNA abundance during the IDC (Fig. 1a). The enrichment analysis reveals that highly expressed genes are more likely to peak their transcription levels during the middle part of the IDC; the late ring through trophozoite and mid schizont stages. In contrast, genes with the lowest expression levels are more likely to peak their transcription at the late schizont stage. This suggests that distinct levels of absolute expression may associate with specific biological functions along the IDC. To evaluate this further, we plotted the level of transcriptional change through the IDC against the maximum mRNA abundance for all expressed genes (Fig. 1b). The overall scatter distribution resembles a “wizard’s hat” with genes tightly clustering together within 83 pathways or functional groups (Fig. 1b and Methods). It reflects that genes with the most dynamic transcription are typically expressed at a very high level at one part of the IDC and strongly suppressed at other (top of the scatter plot). Examples of such genes are factors of merozoite invasion and DNA replication whose transcripts dominate the total mRNA profiles of mid and late schizonts but are highly suppressed in rings and trophozoites (Fig. 1b,c). On the other hand, genes with small fluctuations of transcription across the IDC are expressed at a wide range of levels (bottom of the scatter plot). The highly expressed genes with minimal transcriptional changes include factors facilitating basic metabolic and cellular functions such as translation and protein degradation (Fig. 1b,c). Hence, their metabolic and cellular functions appear to be at high demand regardless the developmental stage. In contrast, genes associated with pyruvate metabolism and intracellular signaling exhibit low expression levels with moderate-to-low changes across the IDC (Fig. 1b,c). The low abundance gene group also involves the *vir* genes whose expression peaks at the extremes of the IDC (young rings or late schizonts, see Supplementary Fig. S5a online). These include 145 of the 170 *vir* genes annotated in the current version of the *P. vivax* genome. However, we observe 25 *vir* genes transcribed at considerably high levels in both isolates (correlation 0.91, see Supplementary Fig. S5b and c online). They are from the phylogenic subfamilies *vir*8, *vir*12, *vir*14, *vir*15 and *vir*16/32 (see Supplementary Table S3 online). This reflects the role of the *vir* genes in antigenic variation of *P. vivax* with transcription of this family being restricted to narrow but distinct subgroups in individual isolates^38^,^39^. Further inspection of the 25 highly expressed *vir* genes reveal two gene clusters at chromosome 2 and 6 (see Supplementary Fig. S5d and e online). The cluster on chromosome 2 contains six *vir* genes of which three (PVX_096970, PVX_096980 and PVX_096987) are adjacent to each other. Notably, these three *vir* genes are expressed consistently throughout the IDC with imperceptible expression variance (bottom 10% percentile). The cluster on chromosome 6 contains four highly expressed *vir* genes that are interspersed by two additional *vir* genes (with low expression) but also by other genes involved in host-parasite interactions including *PHIST*, Duffy Binding Protein (DBP), and a putative exported protein. These additional genes within the chromosome 6 cluster are also expressed at high levels. Until today, little is known about regulatory mechanisms controlling the *vir* genes as well as other factors of the host-parasite interaction. Our data open an intriguing possibility of the existence of transcriptionally active sites in at least two chromosomal locations that control transcription of antigenic determinants in multiple *P. vivax* isolates/clones.

The vast majority of genes encoding basic cellular and biochemical pathways exhibited a significant clustering within the wizard’s hat scatter plot (see Fig. 1b and Supplementary Data S2 online). This suggests a tight co-regulation of transcription within functional pathways that affects not only timing^11^,^31^, but also the overall abundance and dynamics across the IDC (Fig. 1c). Good examples of such co-regulation represent genes of protein metabolism including proteolysis, protein folding, ATP synthesis, posttranslational modifications, chaperone-assisted protein folding and protein processing. The centers of these clusters fell into the middle section of the wizard’s hat which indicates that these pathways are expressed at medium levels and show moderate fluctuations throughout the IDC. There were only few pathways that did not cluster significantly indicating low-to-no co-regulation of their expression (Fig. 1c). This likely reflects divergence in transcriptional regulation within these gene families and thus potentially diversification of their individual functions. These include glycolysis, hemoglobin digestion and the gene family of *AP2*-like transcription factors (Fig. 1c). The *AP*2 genes play a key role in transcriptional regulation of stage specific gene expression during the *Plasmodium* life cycle^40^,^41^ and their temporal patterns of expression are fully conserved between *P. falciparum* and *P. vivax*^11^. However, the diversity of their overall abundance and dynamics may reflect some degree of their functional diversification with some *AP*2 genes functioning during extraerythrocytic development^40^ while others may have acquired a different function such as structural integrity of the telomeres^42^.

### 5′ and 3′ UTRs and TSS

**Figure 2:**
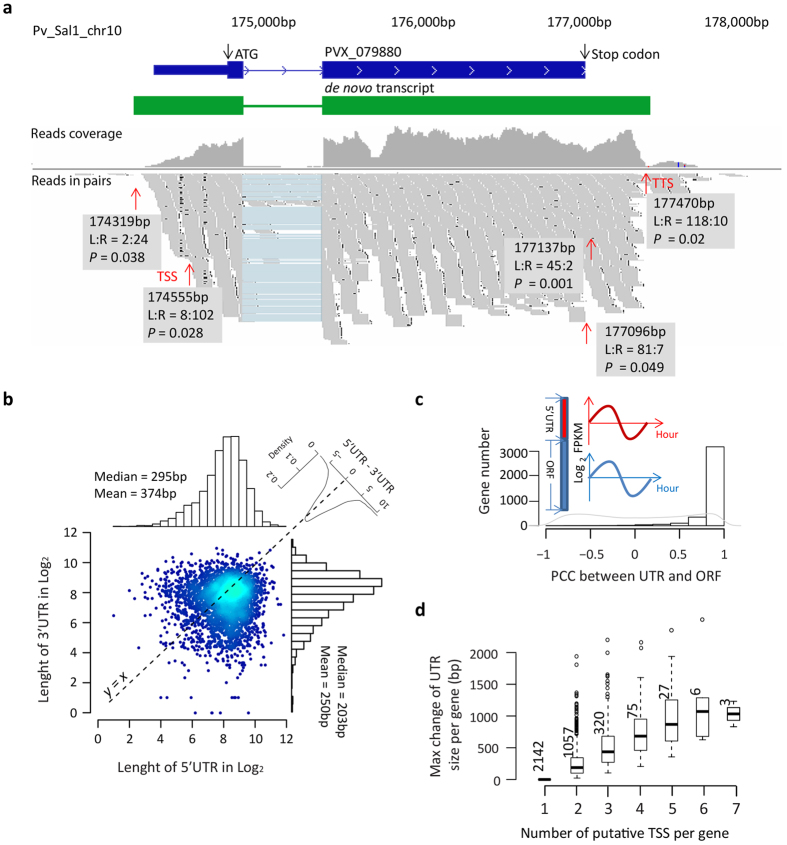
Untranslated regions (UTRs) of the *P. vivax* genes. (**a**) An example of detecting UTR boundaries. The detection of UTR boundary is within the region covered by the *de novo* transcript. Red arrows point out the positions where mapped reads significantly (*P* < 0.05) rise after/before it. The testing window size is 20 bp. For details see Methods. The potential TSS or transcription termination site (TTS) is marked at the position with the highest frequency of starting/ending tags. The position along chromosome, L:R (left:right starting/ending tag) and *P* value at each boundary are listed in grey boxes. (**b**) Scatter plot, histogram distribution of 5′ and 3′UTR length with the median and mean shown inside histograms. The density plot represents the size difference of paired 5′ and 3′UTR of each gene (top corner). The black dash line represents boundaries of equal length of 5′UTR and 3′UTR. The 5′UTR in this diagram represent the most frequent isoform delineated by the main TSS (see Results). (**c**) Histogram of the overall distribution of PCC for transcriptional profiles of 5′UTR (red) and ORF (blue) for 3609 genes. (**d**) The distribution of maximum change of 5′ UTR size in categories of putative TSS number per gene.

To date little is known about both 5′ and 3′ UTRs of *Plasmodium* mRNAs and the vast majority of TSSs remain undetermined. This is particularly a problem for analyses of *cis*-regulatory elements of transcription that is currently based on information from ~20 genes^43^. The higher GC content (~45%) in *P. vivax* genome allows us to use RNA-Seq for a systematic analysis of the UTRs overcoming the problem of AT-rich low complexity sequences that prevented similar studies in *P. falciparum*^28^,^29^. To delineate the 5′ and 3′ UTRs with a particular focus on identifying TSS, we utilized a standard method of *rapid transition of starting/ending tags number* cooperated with the guidance of *de novo* assembled transcripts (Fig. 2a and Methods). For this we combined all the four control reference samples that technically encompass transcripts from all IDC stages from the two individual isolates. Overall, we outlined 5′UTRs and 3′UTRs for 3633 and 3967 genes, respectively (20bp scanning window; *P* < 0.05 Bonferroni corrected *P* value). While the length of the 5′UTRs varied from 0 to 3603bp with a median of 295bp, the 3′UTRs (0–1965 bp, median 203 bp) were significantly shorter (*P* < 2.2e-16) (see Fig. 2b and Supplementary Data S3 online). These results show good correlations with previous studies of *P. vivax* including 1279 5′UTRs and 74 3′UTRs characterized by full length cDNA library sequencing^44^ (see Supplementary Fig. S6a,b online). The transcriptional profile analysis reveals that the UTRs are co-transcribed with their nearest ORF with median PCC 0.96 for SMRU1 and 0.94 for SMRU2 (see Fig. 2c and Supplementary Fig. S6c–f online). This further supports the fact that the identified UTRs belong to the same transcriptional unit together with their adjacent protein coding sequences. As previously suggested^43^,^44^, *Plasmodium* has a substantially longer 5′UTR compared to most eukaryotic species normally represented by a narrow distribution between ~50 bp and 250 bp^45^,^46^ (see Supplementary Table S4 online). In contrast, the *P. vivax* 3′UTRs are typically shorter compared to higher eukaryotes including *Homo sapiens* (886bp), *Mus musculus* (821 bp) and *Danio rerio* (445 bp), but it is still somewhat longer compared to *Saccharomyces cerevisiae* (166 bp)^47^, *Caenorhabditis elegans* (140 bp)^48^ and *Schizosaccharomyces pombe* (203 bp)^49^.

In most eukaryotes, the 5′UTR is shorter than its 3′ counterparts for the majority of genes^50^. Surprisingly, the *P. vivax* genes exhibit an opposite trend (295 bp of 5′UTR versus 203 bp of 3′UTR) (Fig. 2b). To investigate the functional relevance of the UTR size, we applied the Gene Set Enrichment Analysis (GSEA)^51^ to all genes in the rank of UTR size with a cutoff of *P* < 0.01 and *FDR* ≤ 0.25. The result revealed that the longer 5′ UTRs associate with genes coding for proteins in import/export through the nuclear pore (median 396 bp), the membrane of the infected erythrocytes (median 393 bp), mRNA silencing (P bodies, median 384 bp), terpenoid metabolism (median 368 bp) and protein kinases (median 321 bp). The shorter 5′UTRs transcripts are enriched in genes associated with tRNA modifications (median 225 bp) or pyruvate metabolism (median 275 bp). On the other hand, genes encoding proteins of the infected erythrocyte membranes and P bodies have long 3′UTRs with a median size of 227 bp and 268 bp. These functional enrichments suggest a biological significance of the UTRs, possibly in posttranscriptional regulation of gene expression. Moreover the gene functionalities identified by these enrichment analyses represent potential growth limiting/regulating processes and thus in the future, it will be interesting to explore the 5′ as well as 3′ UTRs for the presence of regulatory elements.

**Figure 3:**
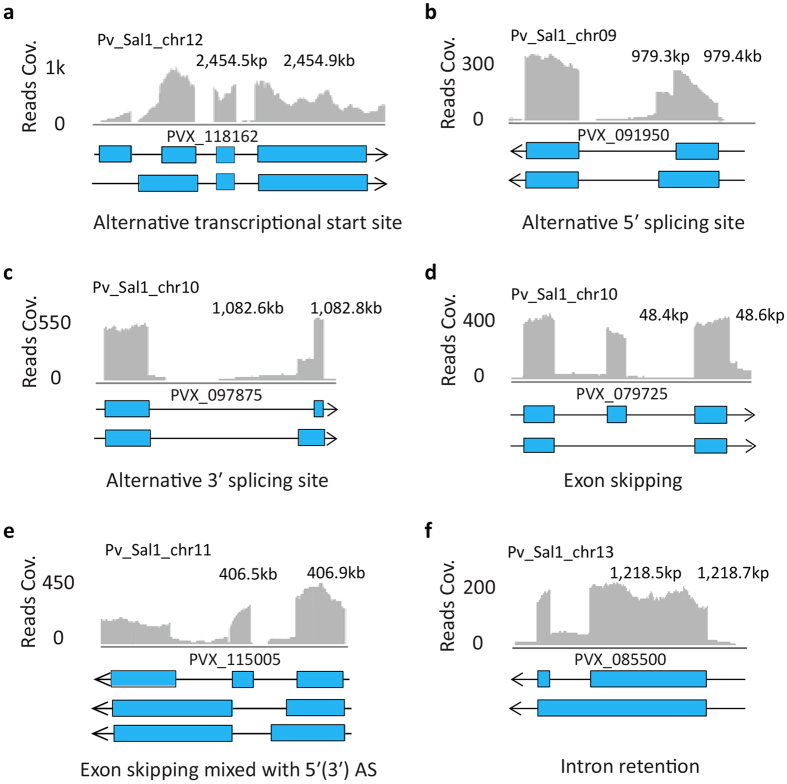
Examples of alternative splicing events in *P. vivax*. (**a**) Exon skipping. (**b**) Alternative 5′ splicing site. (**c**) Alternative 3′ splicing site. (**d**) Exon skipping mixed with 5′(3′) AS. (**e**) Alternative transcription start site with both ends of the first exon altered. (**f**) Intron retention. Blue bars represent exons and the black line introns and the arrow depicts the direction of translation. The histograms above the gene representation depict read coverage (grey bars) as mapped to chromosomal location in the reference genome of *P. vivax* SalI strain (above).

In most eukaryotes, the TSS selection is ubiquitous and facilitates multiple steps of regulation of both mRNA and protein products^52^. In yeast, two or more TSSs are alternatively used by most genes^53^. In human, tissue-specific TSS selection were observed at upstream regions of 6000 ORFs^54^,^55^. In *P. vivax*, we used the method of *rapid transitions of starting tag* and identified multiple TSS for 1491 coding genes (Method, Fig. 2a,d). While the majority of the genes can utilize two or three alternative TSSs (1057 and 320 genes, respectively), up to seven TSSs was detected in at least three genes (Fig. 2d). The multiple TSSs altered the 5′UTRs by 22bp to 2678 bp with higher median value in genes with the higher number of TSSs (Fig. 2d). 32 genes had used different first exons in their transcript isoforms as a result of alternative TSS (see Fig. 3a and Supplementary Table S5 online). Importantly, transcripts starting with minor TSSs display essentially identical temporal transcriptional patterns to those starting with the major (most abundant) TSS. This was shown by normalized counts of starting tags at each TSS within 50 bp window for 849 genes (see Supplementary Fig. S7 online). Altogether, there appears to be a considerable variability of the 5′UTR size due to TSS selection, however, the role of alternative sites of transcriptional initiation does not appear to play a role in life cycle-specific transcriptional regulation given the fact that both the major and minor TSSs share their temporal patterns during the *P. vivax* IDC.

### mRNA splicing

Next, we sought to verify and expand the current annotations of exon/intron boundaries in *P. vivax* by *de novo* transcript assembly. Overall, a total of 8423 putative splicing junctions were identified from the *de novo* transcripts with the false discovery rate (FDR) less than 1% (see Methods and Supplementary Data S4 online). From these, 91.8% (7732) mapped to the annotated open reading frames (ORFs), 5% (432) to 5′UTRs, 0.5% (40) to 3′UTRs and 2.6% (219) fell outside of the current gene models^10^. 137 of the ORF junctions were located outside of the currently annotated introns including 47 junctions which lied at the ORF boundaries. These likely represent previously unidentified introns signifying only small discrepancies in coding start/end positions between the existing gene models and these RNA-Seq results. All the location information of junctions is contained in Supplementary Data S4 online. In addition, we found 61% (84) out of 137 new junctions that were poorly spliced with a splicing efficiency less than 1 (score 1 reflects equal number of spliced and un-spliced reads at a splicing junction) which was calculated based on the sequencing coverage (Methods). 66 of the 84 junctions were completely absent in more than three consecutive time points during the IDC of one or both isolates, and 32 junctions were exclusively present in a single isolate (SMRU1 or SMRU2). Notwithstanding the fact that our observations are based on two Southeast Asian isolates, these results suggest the possibility of stage-specific or isolate-specific splicing events for a limited number of *P. vivax* genes.

### Alternative splicing

These latter findings indicated that AltSpl is present in *P. vivax*, similar to *P. falciparum*^28^, and it plays a role in the *in vivo* growth. In multicellular eukaryotes, AltSpl is a key mechanism for generating transcriptome diversification^56^. However, occurrence of AltSpl in *Plasmodium* appears to be very low as it was only found with no more than 254 *P. falciparum* genes during the entire IDC^28^,^29^. This might contrast with other *Plasmodium* developmental stages such as the sexual development, where up to 16% genes potentially undergo AltSpl^57^. Here, we investigated AltSpl in *P. vivax* by a gene model-independent analysis with clustering of putative splicing junctions and consequently examining shifts between the identified splicing sites (Methods). As a result, we identified 102 AltSpl events (see Supplementary Data S5 online). These include 95 5′ and/or 3′ alternative splicing sites (5′/3′ AltSpl, Fig. 3b,c) and seven exon skipping events of which three are mixed with 5′/3′ AltSpl (Fig. 3d,e). Among the 102 AltSpl events, 63 altered protein coding regions and 39 altered UTRs. Overall the 102 AltSpl events changed transcription products of 2.8% (77 out of 2744) intron-containing genes of *P. vivax* during the IDC. In addition, AltSpl was detected almost exclusively (*P* < 2.2e-16) in highly transcribed genes (see Supplementary Fig. S8a online) and in all cases it exhibits low splicing efficiency with a median score of 0.21. In contrast, the constitutive splice events operate at much higher efficiency (median 3.0). Taken together, the occurrence of AltSpl in *P. vivax* seems as rare as those in *P. falciparum* affecting ~1–2% of the genome^28^,^29^,^57^. Moreover, the vast majority of the alternative transcript isoforms (including those yet to be identified) are likely to occur at low frequencies.

To investigate the stage-specificity of AltSpl during the *P. vivax* IDC, we established the transcription profile of minor transcript isoform (s) for each AltSpl event by counting the number of reads spanning minor splicing junctions at each time point (see Supplementary Fig. S8b–e online). The profile analysis revealed that essentially all abundance peaks of the minor isoforms followed closely the peaks of their corresponding major isoforms in both SMRU1 and SMRU2 time courses. Moreover, the late schizont stage specific genes tend to favor AltSpl mechanism with an overrepresentation (*P* = 0.0122) of 23 genes (for SMRU1 and 21 for SMRU2) among the total of 61 genes which had alternative protein products. This suggests that AltSpl do not regulate timing of gene expression but instead may help in diversifying gene functions, particularly during the late schizont stage. Functional enrichment analysis revealed that the AltSpl in the UTRs are enriched amongst genes encoding S-Glutathionylated proteins (*P* = 0.000137, hypergeometric test) while AltSpl in the coding regions associate with genes encoding nucleic acid binding proteins (*P* = 0.047, hypergeometric test). Although these may be somewhat biased by overrepresentation of highly transcribed genes, the distinct functional groups associated with AltSpl suggests its biological significance in *Plasmodium*. This is supported by a statistically significant overlap between the alternatively spliced gene products in *P. vivax* and *P. falciparum* (see Supplementary Fig. S8f online). In higher eukaryotes, AltSpl is tissue and/or species-specific^58^ and plays a key role in regulating protein expression by nonsense-mediated mRNA decay^59^. In the future studies it will be interesting to investigate whether AltSpl has related functions in *Plasmodium*.

Intron retention is related to AltSpl producing transcripts with un-spliced introns which introduce stop codons or reading frame shifts. Consequently, the resulting transcripts fail to produce functional proteins and as such are redirected into the nonsense-mediated mRNA decay (NMD). In *P. falciparum*, only seven events of intron retention were reported by Otto *et al.*^28^, and only 5.6% introns were estimated to be poorly spliced in the study by Sorber *et al.*^29^. To estimate the frequency of intron retention in *P. vivax*, we used introns confirmed by all four control reference samples and found a similar result to *P. falciparum*. *P. vivax* shows a low frequency of intron retention with 6.5% (530 of 8164) putative introns being retained in transcripts of 421 genes (Fig. 3f). Like AltSpl, intron retention is also rare but distinctly present in *P. vivax* and potentially other *Plasmodia*. This indicates that both of these processes of the RNA metabolism were largely lost throughout the evolution with only a small number of these retained for the asexual blood stage of the *Plasmodium spp*. In future studies it will be interesting to investigate the importance of these RNA processing mechanisms having extremely low levels of occurrence.

### Identification of novel transcripts

**Figure 4:**
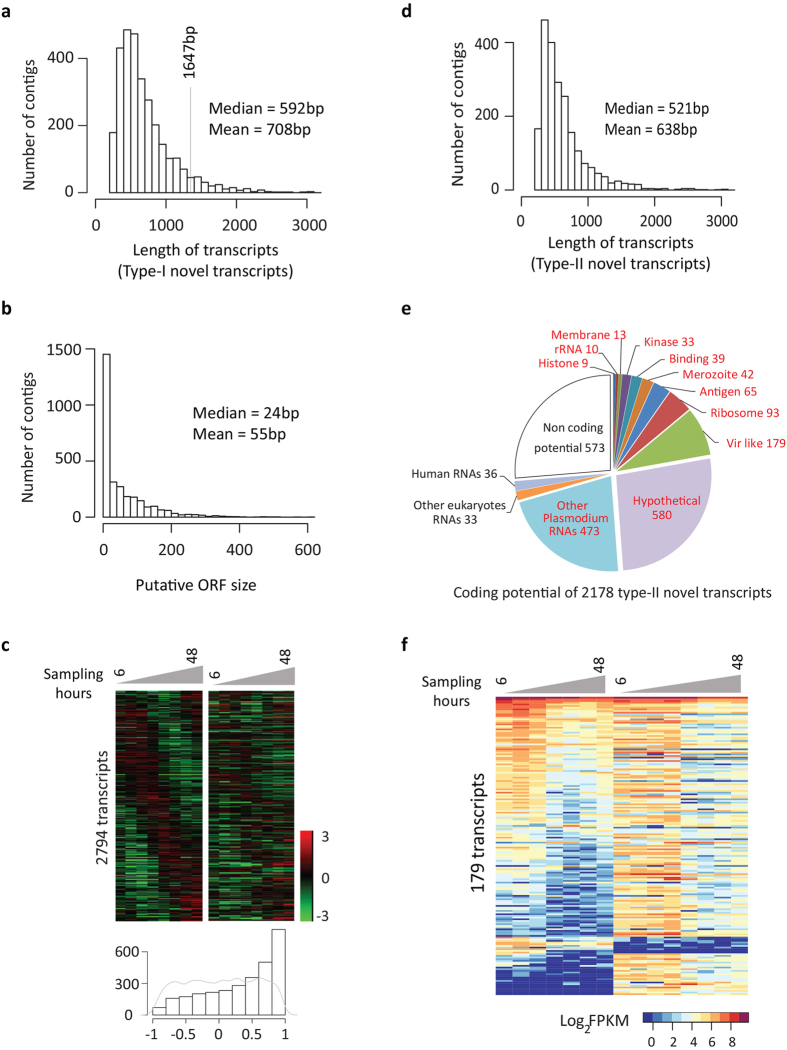
Novel transcripts in the Southeast Asian *P. vivax* strain. (**a**) Length distribution of 3049 type-I novel transcripts which map to genome and outside current gene models. The grey line marks the median size (1647 bp) of protein coding genes currently annotated. (**b**) Length distribution of predicted ORFs for 3018 type-I transcripts without protein homologies. (**c**) Transcriptional profiles in log_2_ ratios for 2794 type-I transcripts (left) in pairs of transcriptional profiles of their nearest downstream gene (right). The histogram on the bottom represents the PCC distribution for paired transcriptional profiles for isolate SMRU2 (see Supplementary Fig. S10 online for SMRU1 data). The grey line represents a PCC distribution of random data. (**d**) Length distribution of 2178 type-II novel transcripts which do not map to current *P. vivax* genome. (**e**) Pie chart of homologous sequences in categories of gene product descriptions for 2178 type-II transcripts. (**f**) Transcriptional profiles in log_2_FPKM for 179 *vir*-like type-II transcripts with SMRU1 data (left) in same order of that with SMRU2 (right).

Next we explored the *de novo* assembled transcripts generated by sequencing of the control reference samples. Here we identified a considerable number of transcripts that were not within the current annotations of the *P. vivax* genome^10^ (Methods). There are two types of novel transcripts: 3049 transcripts that map within the genome but not in any current gene model (type-I) (see Supplementary Data S6 online); 2178 transcripts that do not map to any region within the *P. vivax* or human genomes (type-II) (see Supplementary Data S7 online). The vast majority of the type-I transcripts lack a coding potential. In particular, *in silico* “six frame translations” of 3018 transcripts show no amino acid sequence homologies to any known protein in the NCBI RefSeq database. The type-I transcripts themselves are significantly shorter than the annotated coding genes (median 592 bp versus 1647 bp, Wilcoxon test *P* < 2.2e-16) and the ORFs predicted for these transcripts are extremely short with a median of 24 bp (Methods and Fig. 4a,b). Interestingly, the 3018 type-I transcripts exhibit IDC-dependent transcriptional profiles suggesting that they also undergo stage specific transcriptional regulation (Fig. 4c). Projecting the type-I transcripts to the chromosomes, we find that at least 503 transcripts cluster at 99 distinct loci (sliding window of 4.5 kb was used for binomial test; *P* < 0.05) (see Supplementary Fig. S9 online). Moreover, there are significant correlations of the IDC transcription profile between the type-I transcripts and their nearest downstream coding genes (median PCC 0.51, *P* < 2.2e-16, Wilcoxon rank sum test against random paired profiles) (see Fig. 4c and Supplementary Fig. S10 online). Taken together, the lack of protein coding potential, the nonrandom chromosomal distribution and the IDC dynamics in abundance collectively suggest that the type-I transcripts represent a class of noncoding RNA (ncRNA) in *P. vivax*. ncRNAs have been thought to regulate gene expression that contribute to the subtle differences between closely related species such as humans and other primates^60^. In addition, we found 5.2% (132) putative ncRNAs differentially expressed between the isolates which is 6.5-fold higher than the frequency in protein coding genes. It opens an intriguing possibility that the ncRNAs play a role in strain specific gene expression regulation. On the other hand, there are 31 type-I transcripts exhibiting significantly homologous sequences (size > 100 amino acid and blastx *e*-value < 1*e*-20) to the known *Plasmodium* proteins recorded by RefSeq database. The majority of these belong to the *vir* gene family and other factors of host parasite interactions.

The 2178 type-II transcript contigs do not map to the *P. vivax* genome but are clearly detectable in the RNA-Seq data from all four control reference samples. Although they are as short as type-I, the type-II transcripts are more likely to represent partial (not full length) sequences of coding transcripts (Fig. 4d). This is supported by the fact that 74% (1605) type-II transcripts share significant homologies (size > 100 bp and *e*-value < 1E-20, NCBI blastn against RefSeq mRNAs) with coding sequences including 1536 *Plasmodium* RNAs, 36 human RNAs and 33 other eukaryotes RNAs (Fig. 4e). Amongst the 1536 *Plasmodium* type-II transcripts, 179 are homologous to *vir* genes, 65 to other (than *vir*) antigenic factors and 42 to merozoite invasion genes, 39 to red blood cell binding proteins and 13 putative members of the early transcribed membrane protein (ETRAMP) gene family (see Supplementary Data S7 online). All these gene classes are implicated in host-parasite interactions of *P. vivax* and other *Plasmodia* that are known to be under selective pressure and thus are hyper variable. Hence, we conclude that these type-II transcripts represent Southeast Asia-specific alleles that were not present in the reference genome sequence obtained from the Central American strain SalI^10^. In addition, the 179 new *vir*-like transcripts exhibit transcriptional profiles that are similar to the annotated *vir* genes with a peak expression at the ring-stage (Fig. 4f). This further supports their annotation as new members of this gene family expanding the repertoire of the possible antigenic variation determinants of *P. vivax*. Moreover, 135 of the new *vir* genes are differentially expressed between the two isolates (pairwise Wilcoxon test *P* < 0.05). That is consistent with previous studies showing that *vir* genes expression vary between *P. vivax* strains and isolates as a contributing factor to antigenic variation^9^,^61^,^62^.

In summary, this RNA-Seq analysis of the *P. vivax* IDC provides an in-depth dataset analyzing characteristics of RNA metabolism ranging from temporal and absolute abundance of mRNA to transcript structure including UTRs and splice forms. Moreover, we expand the current annotation of the *P. vivax* genome with a comprehensive list of transcripts that includes mainly genes encoding proteins of host parasite interactions but also non coding RNA transcripts. Importantly this dataset comprises the global transcriptome status of multiple developmental stages of the IDC which allows studying the overall dynamics of the RNA metabolism in *P. vivax*. The presented dataset opens new possibilities into studies of key features of gene expression and with that unique property of *P. vivax* pathophysiology and pathogenesis that will potentially bring new strategies for malaria intervention.

## Methods

### Ethical statement

This study was approved by the relevant local ethics committees and the Oxford Tropical Research Ethics Committee. The methods were carried out in accordance with the approved guidelines. All experimental protocols were approved by the Oxford Tropical Research Ethics Committee and the NTU Institutional Review Board (IRB11/08/03).

### Sample collection and RNA isolation

As we studied the identical RNA samples used in our previous microarray work, the details of sample collection and RNA isolation have been described in the paper^11^. Patients had given informed consent to donate 10 ml of blood for the study. Briefly, the seven sequenced SMRU1 samples were previously labeled as the time point 2 and 4–9 in the paper^11^ corresponding to the 6 hr, 18 hr, 24 hr, 30 hr, 36 hr, 42 hr and 48 hr in *ex vivo* cultures. The eight sequenced SMRU2 samples were previously labeled as the time point 2–9 corresponding to the 6 hr, 12 hr, 18 hr, 24 hr, 30 hr, 36 hr, 42 hr and 48 hr of *ex vivo* cultures. Overall, 250 ng of total RNA per sample was used to construct sequencing library. Four control reference samples were prepared by mixing 250 ng of all time course samples for both SMRU1 and SMRU2 isolates.

### RNA sequencing

These samples were processed with the Illumina TruSeq RNA Sample Preparation Kit v2 following the manufacturer’s recommendations. The libraries were then normalized to 2 nM and validated by qPCR on an Applied Biosystems StepOne Plus instrument, using Illumina’s PhiX control library as the standard. After qPCR validation, the libraries were pooled and sequenced at a final concentration of 11.5 pM across 8 lanes of a HiSeq2000 high-output run at a read length of 100 bp paired end. Overall, we obtained 1.7 billion reads pairs from the 19 sample libraries.

### Mapping and data processing

The sequencing raw reads were aligned to *Plasmodium vivax* Salvador I (SalI) genome^33^ of PlasmoDB^34^ release12 using Tophat2 version2.0.7^63^ with four nucleotides mismatches allowed in each alignment. The parameters were specified as –mate-inner-dist 0 –mate-std-dev 80 -i 10 -I 10000 –library-type fr-unstranded –min-segment-intron 10 –max-segment-intron 10000 -N 4 –read-edit-dist 4. rRNAs Depletion was computationally conducted by removing reads mapping to *P. vivax* r(t)RNA genes that resulted in 205 M reads pairs left to the following study. The proportion of human RNAs was estimated by reads mapping to human RNAs of RefSeq database of Dec 2014. The details are described in Supplementary Fig. S1 and reads number are summarized in Supplementary Table S1 online. All the RNA sequencing raw reads have been deposited into NCBI’s Gene Expression Omnibus^64^ which are accessible through GEO Series accession number GSE61252 (http://www.ncbi.nlm.nih.gov/geo/query/acc.cgi?acc=GSE61252).

### Transcriptome profiles

A total of 101 M uniquely mapped reads pairs (MAQ > 30, probability of miss alignment < 0.001) were used in constructing transcriptional profiles. The proportion of unique reads to the total raw reads ranges from 3.7% to 12.2% with a median of 5.7% per sample. First, the mRNA abundance was measured by the *Fragments Per Kilobase of coding exon per Million fragments mapped* (FPKM) for each protein coding gene at each time point. Second, we filtered out genes with low read coverage (<10 reads) or low abundance (FPKM < 1) in any one of control reference samples. The cutoff selection depends on the standard variation of FPKM values of control reference samples (see Supplementary Fig. S2g online). Third, we fixed the log_2_ FPKM values, which were undetectable; to −4 (the minimum log_2_ FPKM value was −3.16). There were 157 genes with a total of 404 FPKM values not detectable which were 0.52% of the whole data of IDC transcriptome. All the transcriptome data were also deposited into NCBI’s Gene Expression Omnibus which are accessible through GEO Series accession number GSE61252 (http://www.ncbi.nlm.nih.gov/geo/query/acc.cgi?acc=GSE61252).

### Mapping parasite’s sample age to the Plasmodium falciparum IDC

To estimate the age of each isolate time point sample, Spearman's rank correlation coefficient (SRCC) were calculated between global mRNA profiles of syntenic orthologous genes of *P. vivax* and *P. falciparum* for each isolate time point and time points in the reference IDC transcriptome^65^ (every 2 hr sample time point of the *in vitro P. falciparum Dd2* lifecycle). The stage with the peak SRCC value was assigned as the best estimate of the age for parasite collected at each time point (IDC stages shown in Fig. 1a).

### Estimation of timing

The expression timing of a gene was estimated using sine wave function. The expression profile of each gene is modeled using sine function as


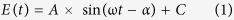


Where 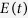
is the log2 ratio of FPKM (sample/control) at the *t* hour of sample collection, *A* is the amplitude of expression profile across life cycle, *C* is the vertical offset of profile from zero, *ω* is the angular frequency, given by *ω = 2π/48* and α is the horizontal offset of profile from zero which we used as phaseogram to show gene expression timing where genes were sorted according to that for transcriptome visualization.

### Differential expression between isolates

A pair wised Wilcoxon test was used to compare transcriptional profiles for each gene between isolates. Significantly differential expression is defined with the cutoff of *P* < 0.05, PCC < 0.5 and the average difference between paired time point of two isolates are less than the maximum difference between corresponding controls. The differential expressed genes are listed in Supplementary Table S2 online.

### Comparisons to microarrays

We reanalyzed the published microarray data^11^ by mapping the old oligos to the *P. vivax* SalI genome of PlasomDB release12. All spots data were subjected to “normexp” background correction followed by lowess normalization within array and quantile normalization between arrays using Limma package of R. Log_2_ ratios of Cy5 over Cy3 intensities were calculated for each spot to represent expression value of a particular probe except those with signal intensity less than 1.5 times the background intensity for both Cy5 and Cy3 fluorescence. For each gene, the expression value was estimated as the average of all probes representing it. Overall, 4989(98%) of 5085 genes designed on microarray display expression profiles without missing values with SMRU1 (5004 or 98% with SMRU2).

To compare RNA-Seq data to microarray data, each expression value (FPKM) were normalized by the average FPKM of controls of the gene and consequently transformed to a log_2_ ratio of FPKM. The transcriptional profiles correlation revealed a complete agreement between RNA-Seq and microarray with the median PCC 0.95 ± 0.06 for 4973 genes with isolate SMRU1 and 0.91 ± 0.1 for 5003 genes with isolate SMRU2. Moreover, the RNA-Seq provides more reliable transcriptional profiles with higher agreement (PCC > 0.5) observed between isolates by RNA-seq to the 304 (80% of 381) genes which showed disagreement (PCC < 0.5) between techniques (see Supplementary Fig. S4b online). In addition, RNA-Seq results improve transcriptional profiles by showing a greater IDC dynamic (max-to-min change) of mRNA abundance which is on average 3-fold (2.8-fold) to the microarray results with isolate SMRU1 (SMRU2).

### Pathway clustering

We applied medoid algorithm to calculate the center of each pathway on the “wizard’s hat” map. In details, for each pathway, we first calculated the dissimilarities matrix for each gene. The dissimilarity between the selected gene and other genes is their Euclidean distance. Next, the medoid gene was chosen as the center one mostly minimizing the sum of dissimilarities matrix. Next, we randomly picked the same number of genes from the “wizard’s hat” for the same pathway and calculated the dissimilarities matrix to the pre-known medoid gene. A Wilcoxon test was performed to compare the observed dissimilarities to random generated dissimilarities. For 738 tested pathways/gene groups, 77(83) in isolate SMRU1 (SMRU2) are significantly (*P* < 0.01) enriched of genes with similar transcription levels and regulation levels which significantly clustered at their medoids on the map of “wizard’s hat” for each. Only the biological process of translation (GO: 0006412) are found with genes significantly scattered on the map (*P* = 0.01) comparing to random data.

### Transcriptome de novo assembly

Full-length transcriptome *de novo* assembly was separately carried out for 28 M uniquely mapped reads and 26 M unmapped reads from control samples using Trinity^66^ of version 2014-07-17. Before input to Trinity, the unmapped reads (unmap to *P. vivax* genome and human RNAs) were trimmed to clean those low quality bases from sequence ends and also clip the remaining adapters using Trimmomatic^67^ of version0.3 with specifying parameters as HEADCROP:9 LEADING:3 TRAILING:3 SLIDINGWINDOW:4:15 MINLEN:30. The outcomes of *de novo* transcripts were aligned to *P. vivax* genome using Blat^68^. We filtered out transcripts matching to *P. vivax* genome region spanning too short (≤200 bp) or too long (≥30 kb) along chromosomes. Finally, the 28 M mapped reads resulted in 21867 to 22563 *de novo* transcripts (mean 22285) per control sample, and the 26 M unmapped reads resulted in 7074 to 10111 *de novo* transcripts (mean 8897) per control sample.

### Detection of UTR and TSS

We detected the UTR boundaries for each protein coding gene using an approach of *rapid transition of starting/ending tags number* similar to previous RNA-Seq studies^45^,^69^. In details, we assumed that, within a transcribed ORF, the number of reads starting/ending at one genomic locus should be similar to that at its neighboring locus. Meanwhile, at the boundary of ORF or the transcriptional start/termination site, the number of starting/ending reads should significantly increase towards the ORF side. Therefore, we applied a normal distribution model to the starting/ending reads number at genomic positions within transcribed exons:


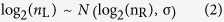


Where n_L_(n_R_) is the number of starting/ending reads in 20 bp window on the left(right) side of a genomic position, and σ equal to 1 which was estimated based on calculation using 10000 random selected positions within ORF exons. Next, for each upstream position of the start codon and downstream position of the stop codon within the region covered by the longest *de novo* assembled transcript which belonged to the studied gene solo, we tested the probability it falls into the null hypothesis and the UTR boundary (or TSS) was set at the position which refused the null hypothesis at *P* ≤ 0.05. For genes with multiple positions passing the cutoff *P* ≤ 0.05, UTR boundary was set to the only position with the highest frequency of starting/ending reads. To sharp the UTR boundaries, we merged reads alignment from all four control references.

### Splicing junctions

The putative splicing junctions or introns are defined independently from the current gene models. We aligned the *de novo* transcripts to the genome of *P. vivax* SalI and extracted all junctions spanning at least 10 and no more than 1 k nucleotides along the genome sequence. The putative splici junctions required to be detectable by three or more controls and contain canonical splicing sits of donor/acceptor sequences (GT/AG) at both ends. Overall, a total of 8423 putative splicing junctions (putative introns) are detected. Next, we estimated the false discovery rate (FDR) for each of 8423 splicing junctions. The positive dataset is the 8423 splicing junctions. The negative dataset were constructed by those putative splicing junctions without known splicing site sequences, GT-AG or GC-AG^29^, at either ends. The FDR was calculated as:


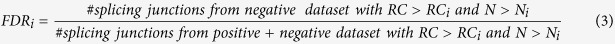


Where *RC*_*i*_ is the total read counts from four control references confirming the *i*th junction, and *N*_*i*_ is the observing time of the *i*th junction by control references. All the 8423 splicing junctions passed the cutoff of FDR < 1%.

### Splicing efficiency

The splicing efficiency of a splicing junction was measured by the number of reads spanning that junction with more than 10 bp matching to each flanking sequences over the average number of un-spliced reads at the “exon-intron” sites on both sides^46^.

### Detection of alternative splicing

The Altspl events were also determined independently from current gene models. First, the putative splicing junctions were grouped by their locations. In practice, two junctions were grouped together if their locations overlapped to each other, and two groups were merged if any member of them overlapped to each other. Subsequently within each group, Altspl events were characterized into distinct type such as alternative 5′/3′ splicing sites, exon skipping or mixture of both types (Fig. 3).

To compare Altspl between time points and isolates, the transcription level of minor isoform was estimated by the number of reads spanning the minor splicing junction followed by control normalizing it to obtain log_2_ ratios of FPKM.

### Identification of novel transcripts

For the incompleteness of *P. vivax* genome map, we perform *de novo* assembly for mapped reads and unmapped reads separately and characterized the result of *de novo* transcripts into two categories: type-I *de novo* transcripts reconstructed by mapped reads and aligned to genome regions outside current gene models; type-II *de novo* transcripts reconstructed by reads unmapped to *P. vivax* or human RNAs. To reduce the data redundancy, we clustered *de novo* transcripts into groups and use the longest transcript of each group to represent one cluster. A cluster is established if transcript “a” from control “A” is the reciprocal best hit of transcript “b” from control “B” by BLAT searching for > 100 bp nucleotides matched to each other. Clusters are merged if any members of them satisfy the same criteria above. The final novel transcripts required reproducible reconstructions in each controls. Finally, we identified 3049 type-I novel transcripts (2890 from chromosome and 159 from super contigs AAKMs) and 2178 type-II novel transcripts.

The transcriptional profiles of each novel transcript are established based on the transcription levels of corresponding cluster. First, we align reads used for transcriptome assembly against *de novo* transcripts using BWA^70^. Second, the read counts of each transcript cluster at a particular time point were normalized by the size of the longest transcript of that cluster and the total number of mapped reads of that time point. Finally, transcriptional profile of each novel transcript are established using normalized read counts and displayed in the order of *Phaseogram* (Fig. 4c,f). For type-I novel transcripts mapping to intergenic genome regions, the temporal pattern of transcriptional profile is shown as the log2 ratio of normalized read counts of each time point to average controls (see Fig. 4c and Supplementary Fig. S10 online).

### Estimating ORF size for type-I novel transcripts

The possible ORF is the longest putative coding region starting from an “ATG” codon and terminating at anyone of the stop codon of TAG, TAA or TGA.

### Functional annotation and homology searches

We annotated *P. vivax* genes according to their homologous genes in *P. falciparum* which has been functionally annotated by the database of Kyoto Encyclopedia of Genes and Genomes (KEGG)^71^, Malaria Parasite Metabolic Pathways (MPM)^72^ or Gene Ontology (GO)^73^ till Jan 2015. The homologs information was downloaded from PlasmoDB^34^. By this approach, 816 *P. vivax* genes were annotated onto KEGG pathways, 2590 genes onto MPM pathways and 3944 genes onto GO terms. To search for the homologous proteins of the novel transcripts, we used the tool of NCBI Blastx (http://blast.ncbi.nlm.nih.gov/Blast.cgi) against NCBI Protein Reference Sequences^74^.

## Additional Information

**How to cite this article**: Zhu, L. *et al.* New insights into the *Plasmodium vivax* transcriptome using RNA-Seq. *Sci. Rep.* **6**, 20498; doi: 10.1038/srep20498 (2016).

## Supplementary Material

Supplementary Information

Supplementary Dataset 1

Supplementary Dataset 2

Supplementary Dataset 3

Supplementary Dataset 4

Supplementary Dataset 5

Supplementary Dataset 6

Supplementary Dataset 7
