# Supplementary material for: New insights into the Plasmodium vivax transcriptome using RNA-Seq
Source: Sci Rep. 2016 Feb 9;6:20498. doi: 10.1038/srep20498 (PMC4746618; doi:10.1038/srep20498)
Supplement: Supplementary Information [file srep20498-s1.pdf]

New insights into the *Plasmodium vivax* transcriptome using RNA-Seq

Lei Zhu[1], Sachel Mok[1], Mallika Imwong[2], Anchalee Jaidee[3], Bruce Russell[4], Francois Nosten[3], Nicholas P Day[2], Nicholas J White[2], Peter R Preiser[1,*] and Zbynek Bozdech[1,*]

[1]School of Biological Sciences, Nanyang Technological University, Singapore 637551;

[2]Wellcome Trust Mahidol University Oxford Tropical Medicine Research Programme;

[3]Sholklo Malaria Research Unit, Faculty of Tropical Medicine Research, Mahidol University, Salaya, Nakhon Pathom 73170, Thailand; [4]Laboratory for Malaria Immunology, Singapore Immunology Network, Biopolis, Agency for Science, Technology and Research, Singapore 138632;

[*]Corresponding authors: Zbynek Bozdech and Peter R Preiser

To whom correspondence should be addressed at: School of Biological Sciences, Nanyang Technological University, 60 Nanyang Drive, Singapore 637551. Telephone: +65-63162925/63162822. Email: ZBozdech@ntu.edu.sg, PRPreiser@ntu.edu.sg

SUPPLEMENTARY INFORMATION

Supplementary Figure S1. Workflow of data processing and *de novo* transcriptome assembly.

Supplementary Figure S2. The reproducibility of control reference samples and cutoff selection for expressed genes.

Supplementary Figure S3. IDC transcriptome of SMRU1.

Supplementary Figure S4. Comparison of RNA-Seq and microarray data.

Supplementary Figure S5. Transcription and regulation of vir genes of *P.vivax*.

Supplementary Figure S6. Histograms of Pearson Correlation Coefficient of transcriptional profiles between UTR and their nearest coding sequence (CDS).

Supplementary Figure S7. Transcriptional profiles of genes having TSS choices.

Supplementary Figure S8. Expression of genes with Alternative Splicing (AS).

Supplementary Figure S9. Chromosome projection of 3049 ncRNA-like transcripts.

Supplementary Figure S10. Expression correlation between type-I transcripts and their nearest downstream genes.

Supplementary Table S1. RNA-Seq reads mapping statistics against *P.vivax* genome.

Supplementary Table S2. Differentially expressed genes between isolates.

Supplementary Table S3. List of 25 *vir* genes highly expressed in both SMRU1 and SMRU2.

Supplementary Table S4. UTR size of selected species from Refseq database.

Supplementary Table S5. Genes with TSS selection confirmed by *de novo* transcript isoforms.

Supplementary Data S1. IDC transcriptome

Supplementary Data S2. Pathways clustering

Supplementary Data S3. UTRs

Supplementary Data S4. Junctions

Supplementary Data S5. AltSpl events

Supplementary Data S6. TypeI novel transcripts

Supplementary Data S7. TypeII novel transcripts

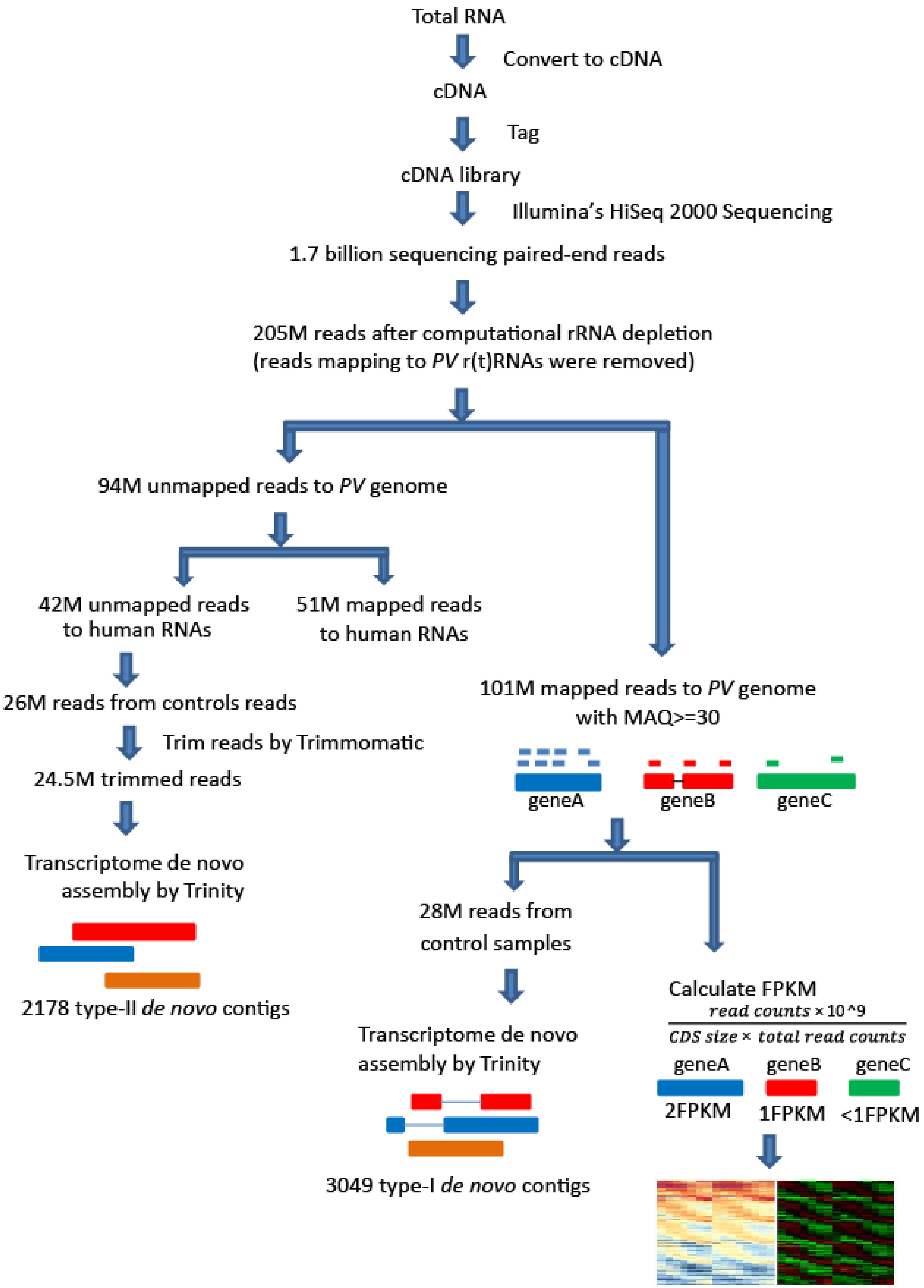

Supplementary Figure S1. Workflow of data processing and *de novo* transcriptome assembly.

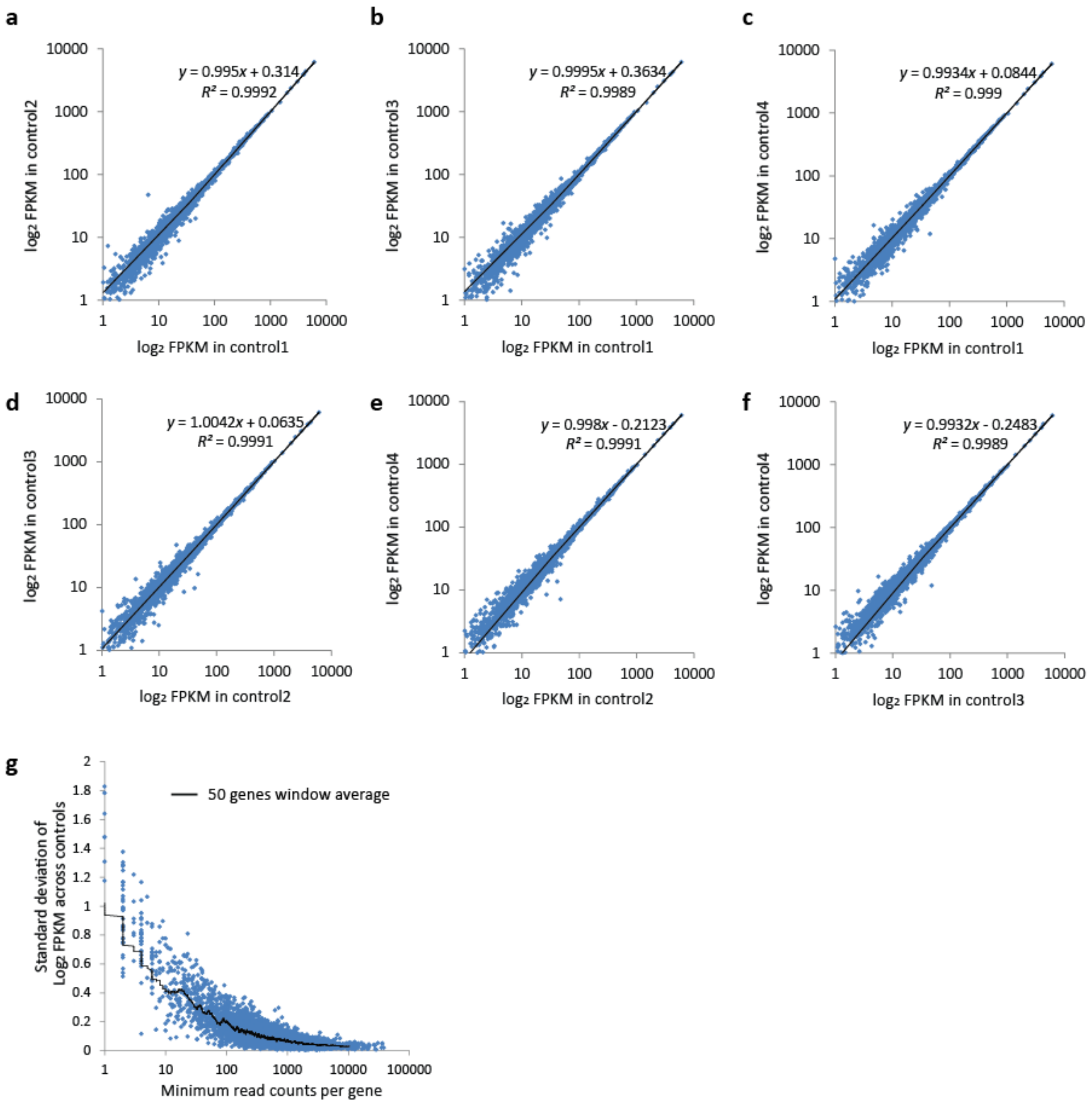

Supplementary Figure S2. The reproducibility of control reference samples and cutoff selection for expressed genes. (a-f) Scatter plots of gene expression levels in log2 FPKM of the control samples across all pair-wise comparisons. (g) Scatter plot of standard deviation of gene expression across control reference samples against read counts per gene.

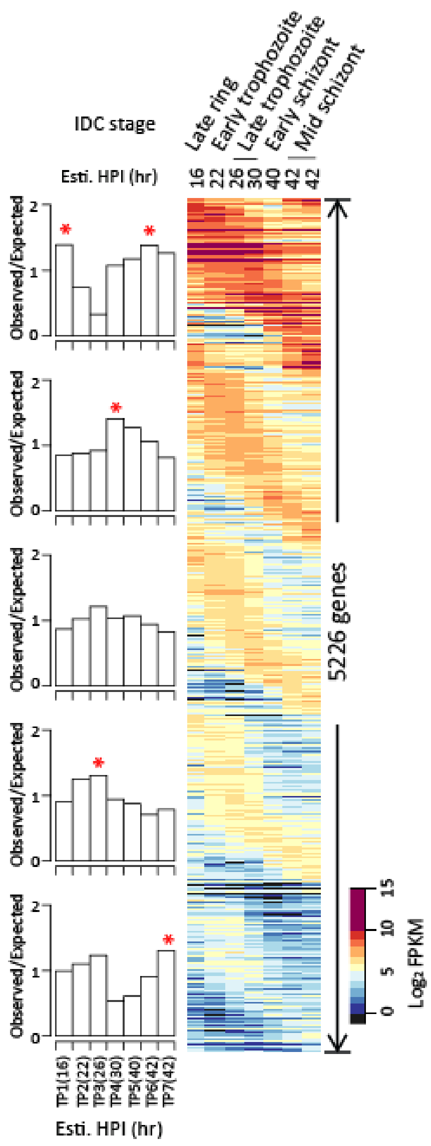

Supplementary Figure S3. IDC transcriptome of SMRU1. The heat map shows mRNA abundance (log_2FPKM) of 5226 annotated protein-coding genes across IDC in the same order as shown in Fig. 1a. Left bar plots represent the fold enrichment of time-point specific genes (genes peaking their transcription at the same particular time point during the IDC) for each group. The expected frequency used here is the proportion of genes maximally expressed at the time point in whole genome; * indicates over-representation by binomial test at *P*<0.01

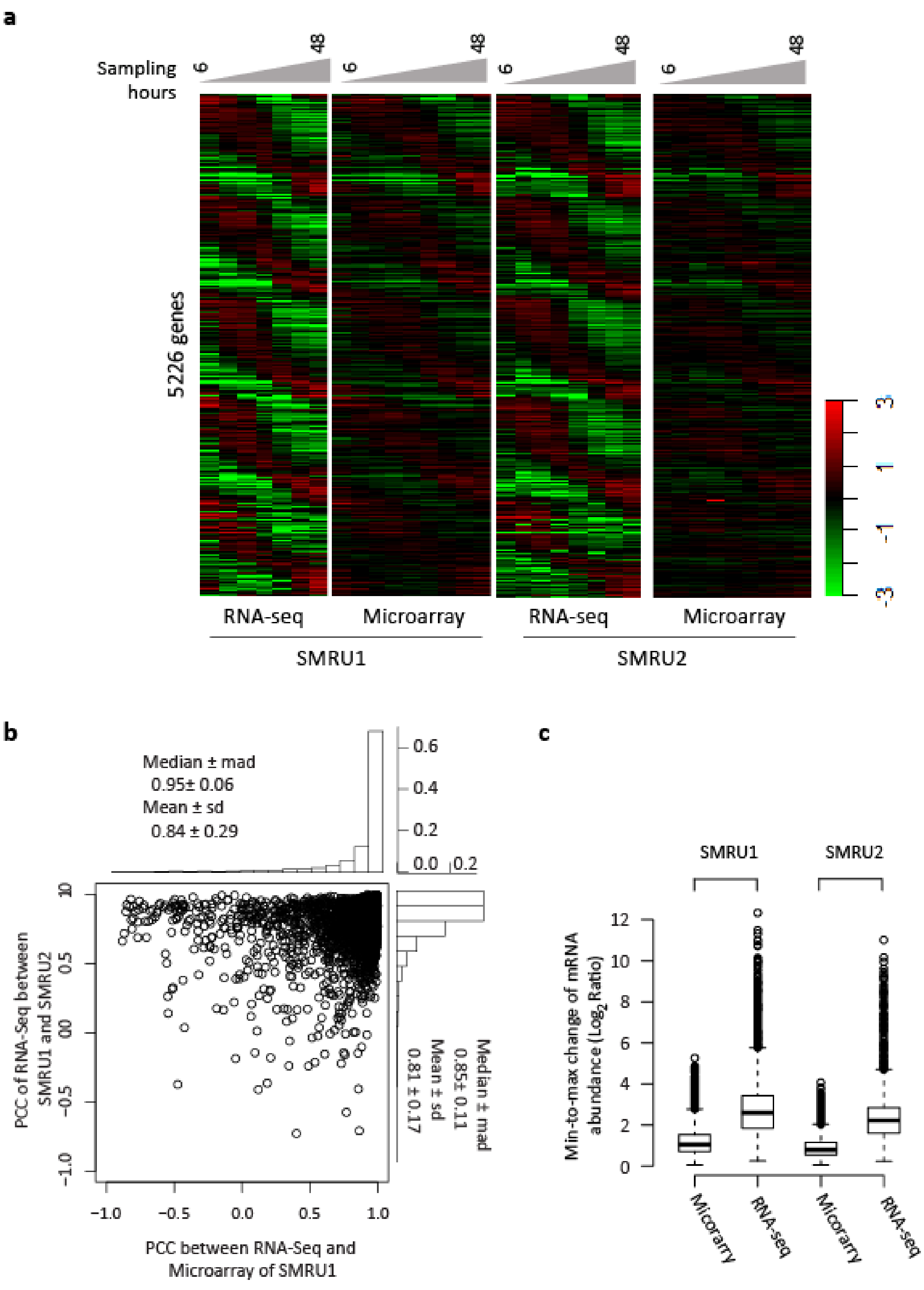
a
Sampling hours
6
48
5226 genes
RNA-seq
Microarray
SMRU1
RNA-seq
Microarray
SMRU2
3
1
-1
-3
b
Median ± mad
0.95± 0.06
Mean ± sd
0.84 ± 0.29
0.6
0.4
0.2
0.0
0.2
Median ± mad
0.85± 0.11
Mean ± sd
0.81 ± 0.17
PCC of RNA-Seq between SMRU1 and SMRU2
PCC between RNA-Seq and Microarray of SMRU1
-1.0
-0.5
0.0
0.5
1.0
c
SMRU1
SMRU2
Min-to-max change of mRNA abundance (Log2 Ratio)
12
10
8
6
4
2
0
Micorarry
RNA-seq
Micorarry
RNA-seq

d

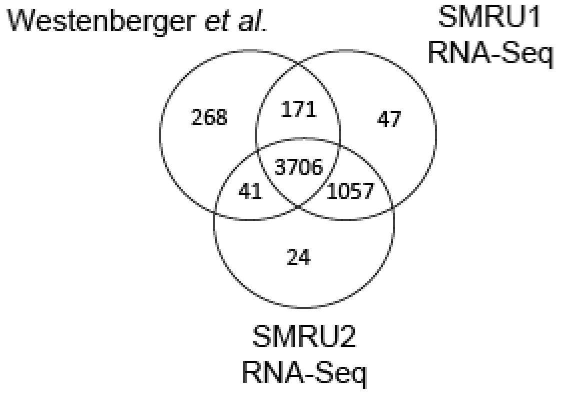

e

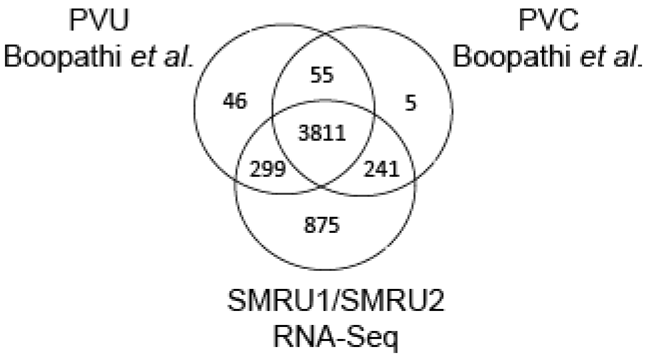

Supplementary Figure S4. Comparison of RNA-Seq and microarray data. (a) Transcriptional profiles for 5226 genes in the same order as shown in Fig. 1a and Supplementary Fig. S3. For each gene, the expression level at a given time point is derived from $\log_2$ratios of mRNA abundance (FPKM) at that time to the average controls of that gene. (b) Scatter plot and histogram of overall PCC distributions between isolates and methods (RNA-Seq and microarray). (c) The boxplot show the comparison of max-to-min change of mRNA abundance ($\log_2$ ratio) during IDC between methods for isolate SMRU1 and SMRU2. (d) Venn diagram of genes changing their overall expression by more than two fold across the IDC in the presented RNA-Seq study and the Westenberger's study[12]. (d) Venn diagram of genes with representative expression in the presented RNA-Seq study and the expression of Boopati's study[37].

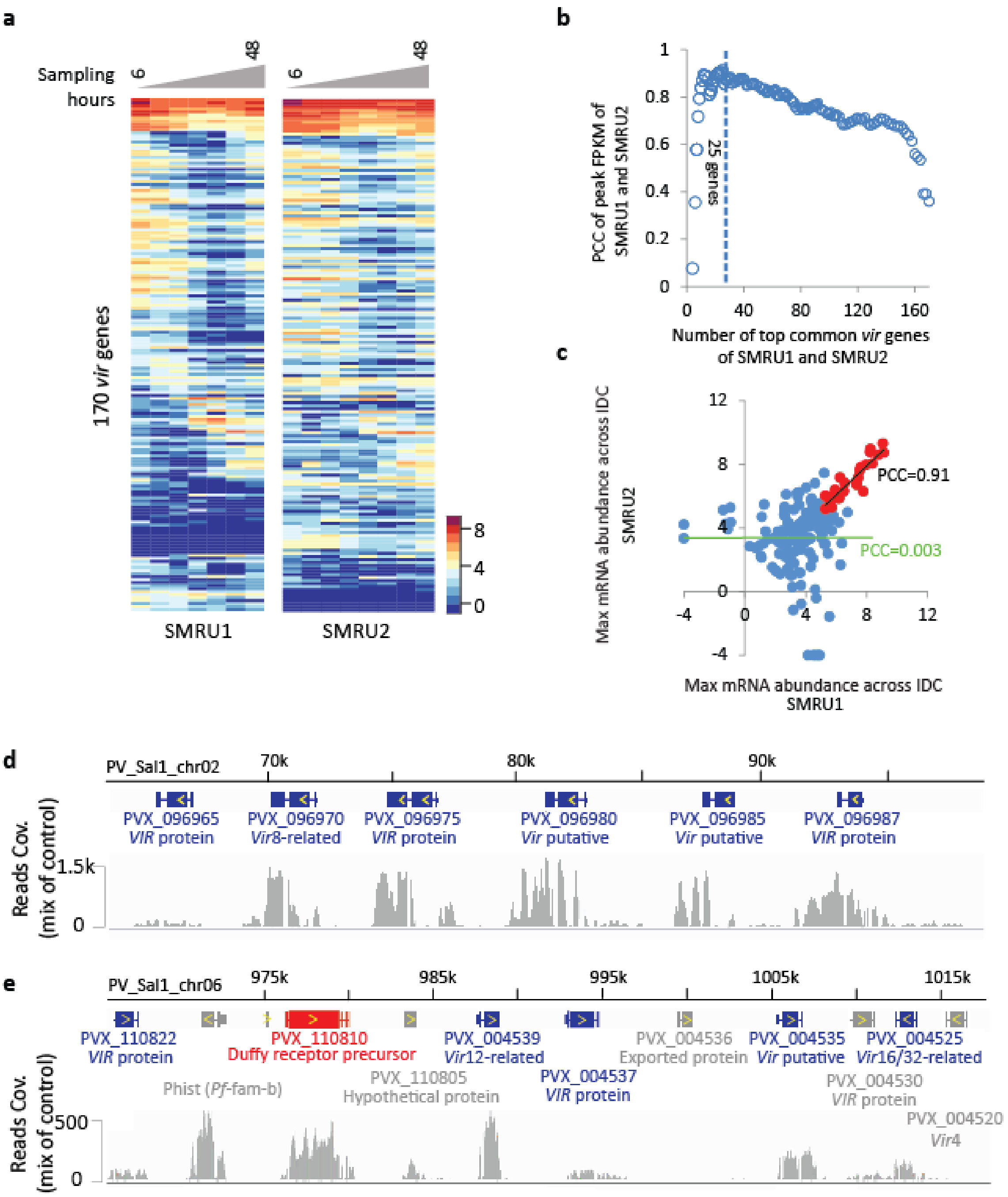

Supplementary Figure S5. Transcription and regulation of *vir* genes of *P. vivax*. (a)Transcriptional profiles in log_2FPKM of 170 *vir* genes for isolate SMRU1(left) and SMRU2(right). (b) Classification of *vir* genes based on their peak expression level (FPKM) in SMRU1 and SMRU2. The dash line indicates the cutoff at top 25 genes (red dots in c) which show the highest correlation (PCC=0.91) of peak expression between two isolates. Blue dots in c represent genes lowly expressed in SMRU1 or/and

SMRU2 which show very low correlation (PCC=0.003). The black and green line represents linear regression based on red and blue dots respectively. (d) *Vir* genes cluster on chromosome 2. Blue box on the top row represents coding regions of annotated genes within the shown region captured from IGV browser. The yellow arrows show the transcriptional direction of individual genes. Peaks in grey at the bottom row represent reads coverage based on the mix data of controls (e) *Vir* genes cluster on chromosome 6. Blue boxes represent *vir* genes highly expressed in both isolates. Grey boxes represent their neighbor genes and Red boxes highlight the gene of Duffy receptor precursor.

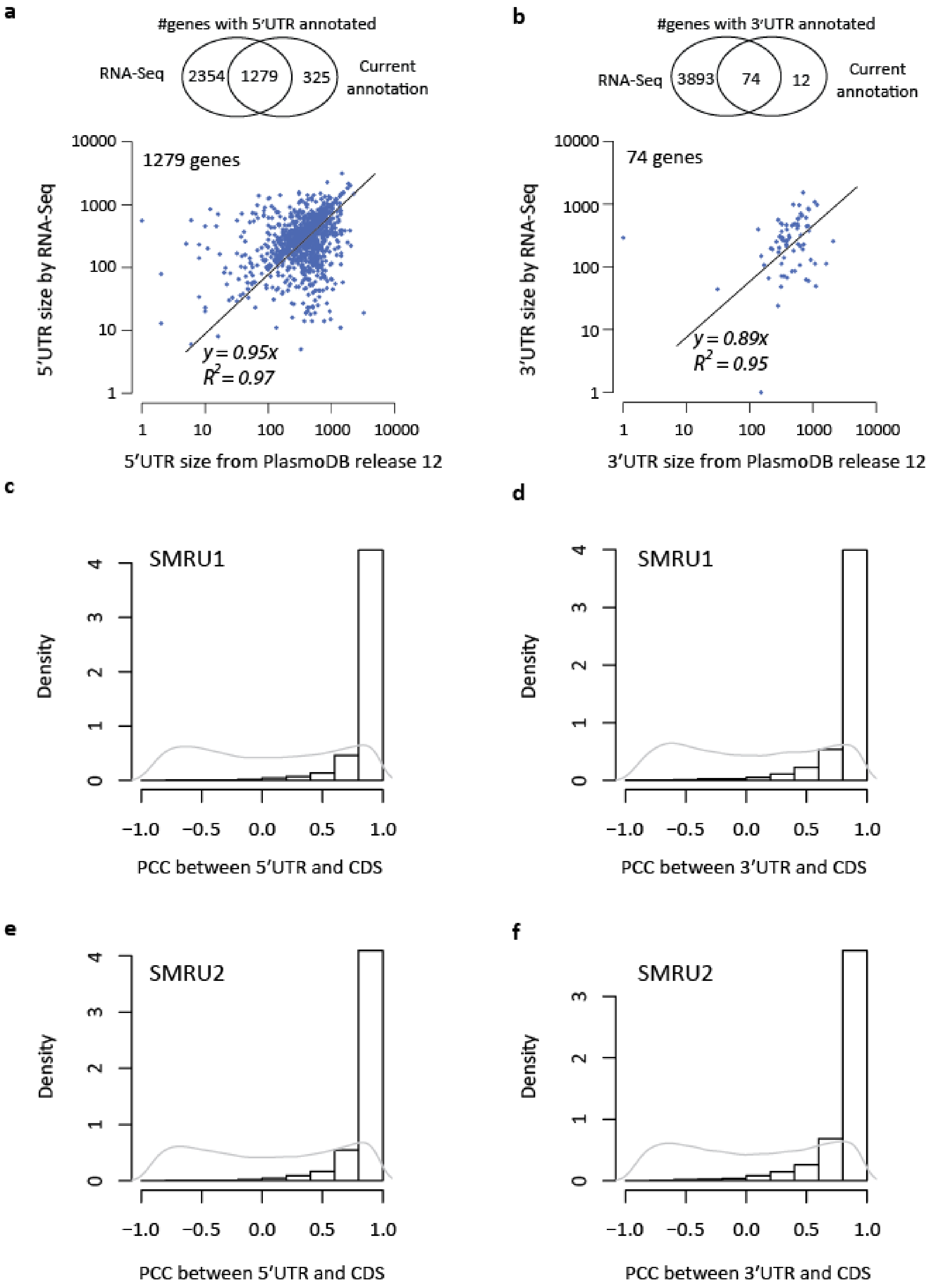
a
#genes with 5'UTR annotated
RNA-Seq
2354
1279
325
Current annotation
1279 genes
y = 0.95x
R2 = 0.97
5'UTR size by RNA-Seq
5'UTR size from PlasmoDB release 12
b
#genes with 3'UTR annotated
RNA-Seq
3893
74
12
Current annotation
74 genes
y = 0.89x
R2 = 0.95
3'UTR size by RNA-Seq
3'UTR size from PlasmoDB release 12
c
SMRU1
Density
PCC between 5'UTR and CDS
d
SMRU1
Density
PCC between 3'UTR and CDS
e
SMRU2
Density
PCC between 5'UTR and CDS
f
SMRU2
Density
PCC between 3'UTR and CDS

Supplementary Figure S6. Histograms of Pearson Correlation Coefficient of transcriptional profiles between UTR and their nearest coding sequence (CDS). (a) Compare the RNA-Seq derived 5 'UTRs to the current annotation from PlasmoDB. (b) Compare the RNA-Seq derived 3 'UTRs to the current annotation from PlasmoDB. The histograms of density show the distributions of PCC for 3609 5'UTRs(c), 3908 3'UTR (d) and their nearest CDS of isolate SMRU1 and SMRU2 (e and f) respectively. The grey line represents PCC distribution generated by datasets of random paired UTRs and CDSs.

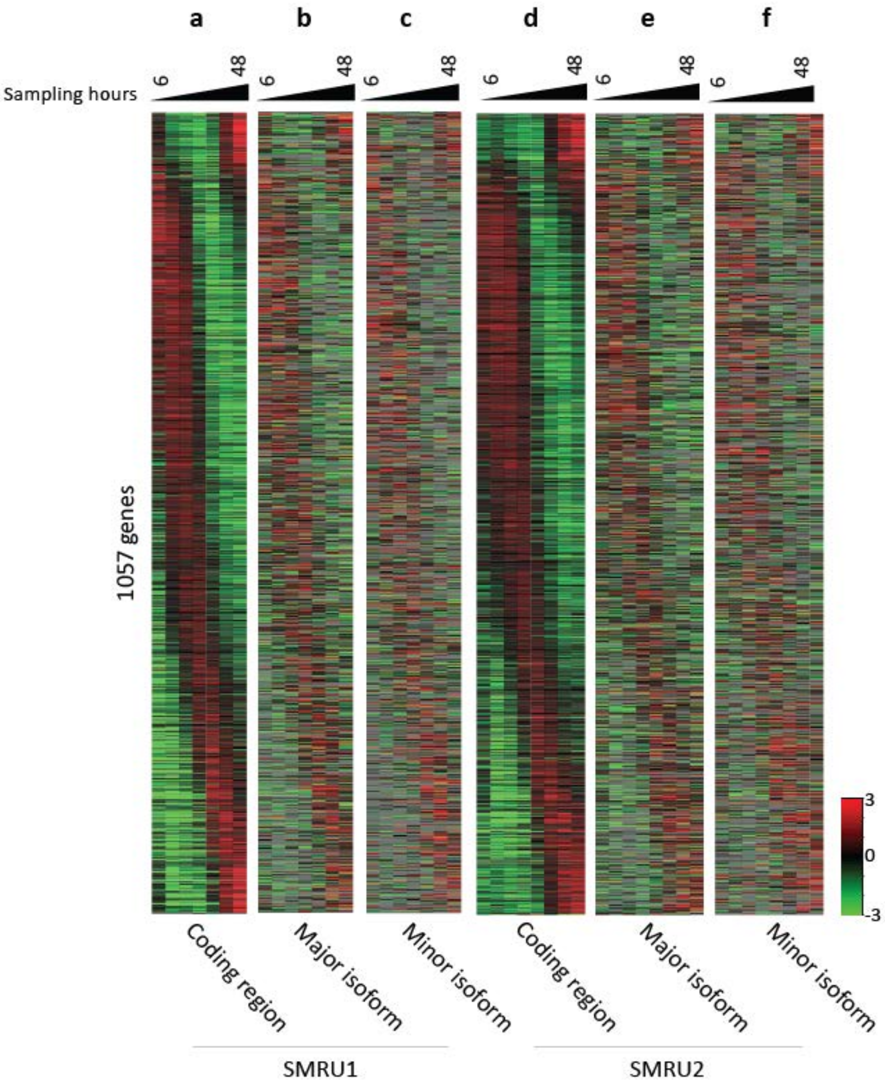

Supplementary Figure S7. Transcriptional profiles of genes having TSS choices. Transcriptional profiles of 1057 genes with two putative transcription start sites (TSSs) based on expression level (log_2ratios) of their coding regions (a&d), isoforms with major TSSs (b&e major isoform) and isoforms with minor TSSs (c&f minor isoform) for SMRU1 and SMRU2 respectively. The expression level of each isoform at a given time point is estimated by starting read counts within the 50bp window downstream TSS which is consequently normalized by the library size and average controls of that time point.

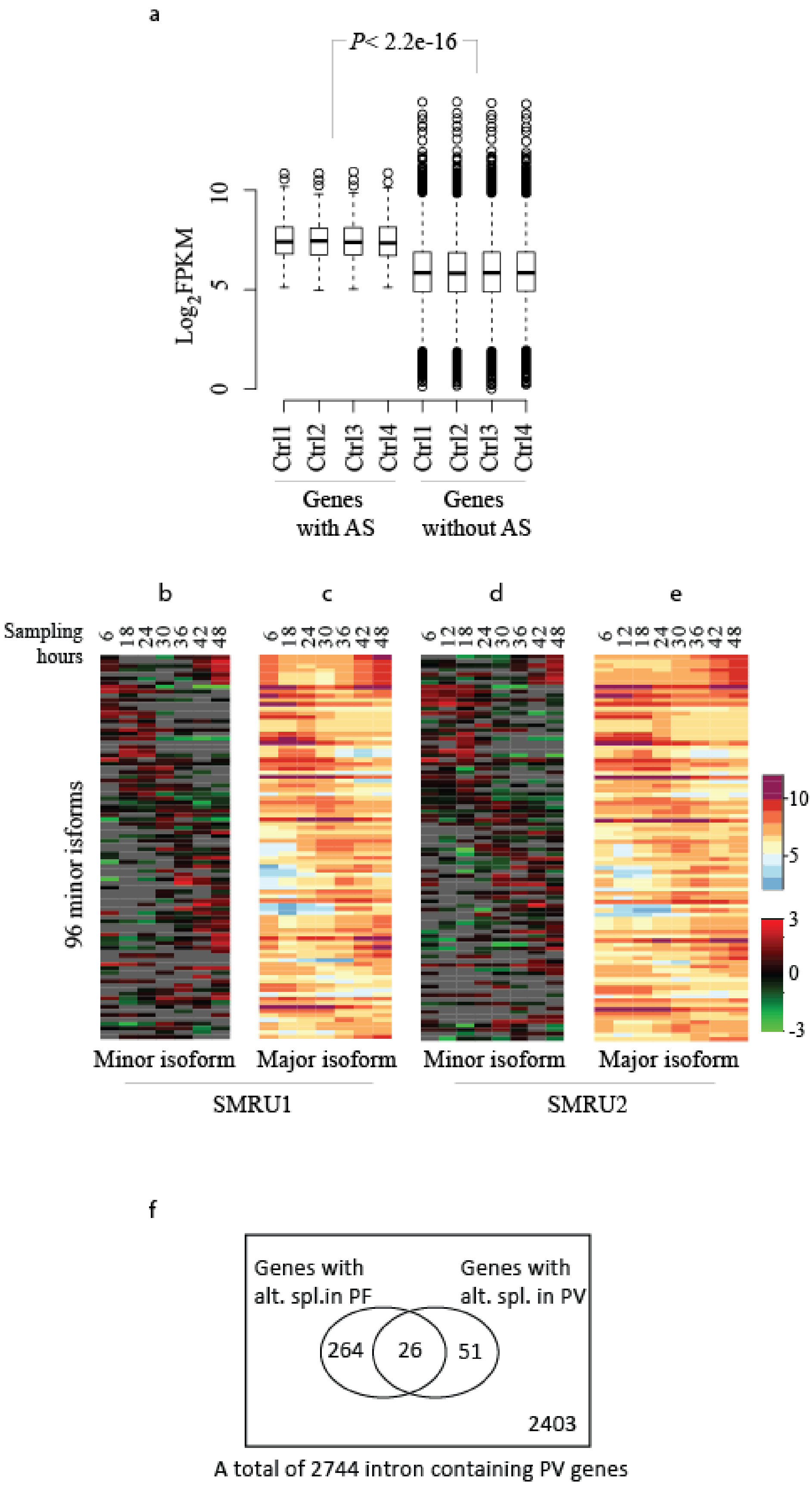
a
P< 2.2e-16
Log2FPKM
0
5
10
Ctrl1
Ctrl2
Ctrl3
Ctrl4
Ctrl1
Ctrl2
Ctrl3
Ctrl4
Genes with AS
Genes without AS
b
c
d
e
Sampling hours
6 18 24 30 36 42 48
6 18 24 30 36 42 48
6 12 18 24 30 36 42 48
6 12 18 24 30 36 42 48
96 minor isforms
10
5
3
0
-3
Minor isoform
Major isoform
Minor isoform
Major isoform
SMRU1
SMRU2
f
Genes with alt. spl.in PF
Genes with alt. spl. in PV
264
26
51
2403
A total of 2744 intron containing PV genes

Supplementary Figure S8. Expression of genes with Alternative Splicing (AltSpl). (a) Boxplot of expression levels in categories of genes with and without AltSpl events according to control references from the 1st (Ctrl1) to the 4th (Ctrl4). The result shows alternatively spliced gene products expressed in significantly higher level comparing to those constitutively spliced gene products (P<2.2e-16). (b-e) Transcriptional profiles of alternatively splicing genes (d&f) and their minor transcript isoforms (c&e) for isolate SMRU1 and SMRU2. For each minor transcript isoform of 80 genes with AltSpl, the mRNA abundance at a given time point is expressed as the number of reads spanning that isoform-specific splicing junction and the read counts are normalized by library size of that time point. The transcriptional profiles of the 96 minor isoforms are established using log_2 ratios of mRNA abundance at each time point to the average control reference. The transcriptional profiles of major isoforms are established using log_2FPKM of the annotated coding sequences of corresponding genes like Fig. 1a shown. Grey colors represent 42% and 38% missing data from the time course samples of SMRU1 and SMRU2 respectively. 77% (79%) of the minor splicing show representative values or maximum values near the peak of major transcripts. (f) Venn diagram of genes with alternatively spliced products (alt. spl.) in *P. falciparum* and *P.vivax*. To compare the AltSpl events of *P.falciprum* to *P.vivax*, we merged the result from the published work of *Otto et al.*[28] and Sober *et al.*[29]. The resulted dataset of 299 AltSpl *P.falciprum* genes correspond to 290 orthologous genes in *P.vivax* which is shown as the number of “genes with alt. spl.in PF” in the Venn diagram.

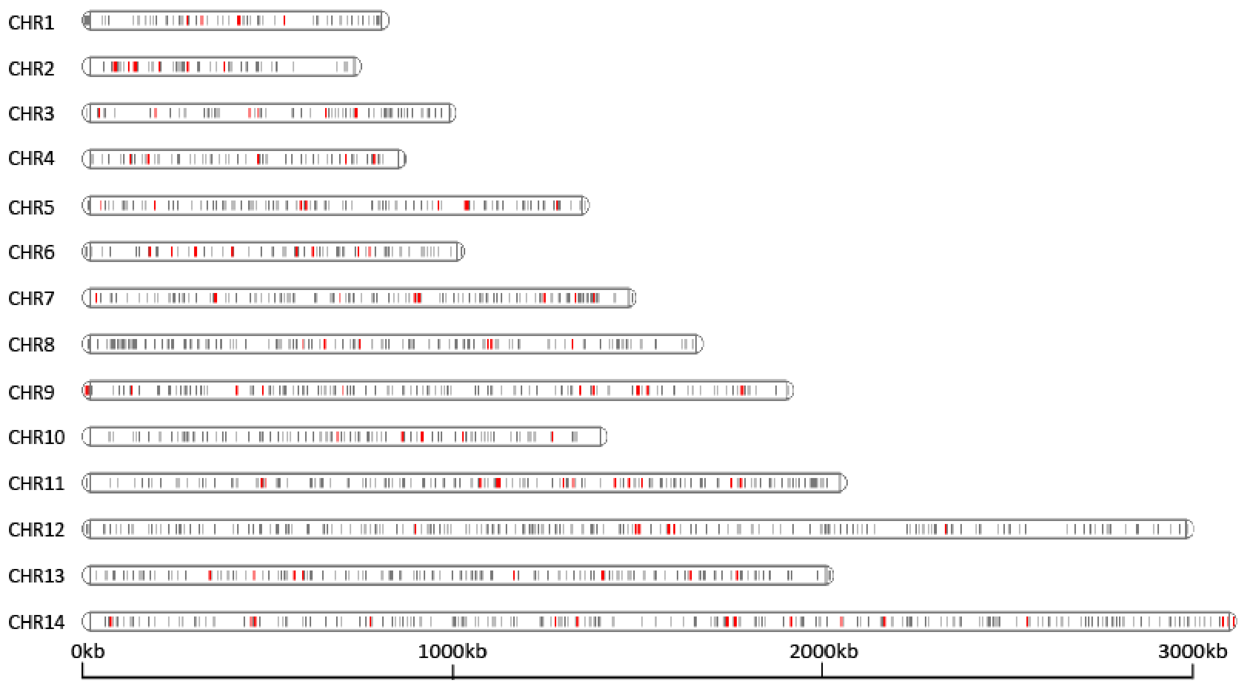

Supplementary Figure S9. Chromosome projection of 3049 ncRNA-like transcripts. Red bars represent 99 clusters of 503 transcripts plotted along the 14 chromosomes.

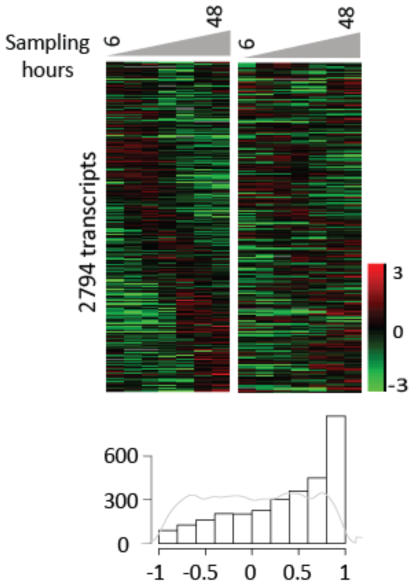

Supplementary Figure S10. Expression correlation between type-I transcripts and their nearest downstream genes. Transcriptional profiles in Log_2ratios for 2794 type-I transcripts (left) and the nearest downstream gene of each in the same order of peak expression time (right). The histogram on the bottom represents the distribution of PCC for transcriptional profiles of each pair of the type-I transcript and its nearest downstream gene with SMRU1 (see Fig. 4c for SMRU2 data). The grey line represents a randomly generated PCC distribution.

Supplementary Table S1. RNA-Seq reads mapping statistics against *P. vivax* genome

Sample	Sampling hour	Total reads	Reads mapping to r(t)RNA	Reads mapping to human RNAs(%)	Reads uniquely mapping to P. vivax (MAQ>30)		Trimmed &unmapped reads	Avg. reads coverage [a]
					Super contigs	Chromosomes I-XIV		
SMRU1	6hr	76,836,159	53,866,902	11,080,911 (14.4%)	6,935,138	2,403,433	970,731	21
	18hr	83,472,491	75,489,415	2,084,041 (2.5%)	1,468,593	3,061,533	854,240	27
	24hr	74,642,345	69,077,255	969,242 (1.3%)	719,064	2,773,344	785,287	25
	30hr	68,001,824	63,640,665	634,963 (0.9%)	493,365	2,219,188	835,532	20
	36hr	81,000,381	76,119,553	610,057 (0.8%)	493,193	2,614,901	943,300	23
	42hr	81,564,809	76,633,221	769,203 (0.9%)	626,924	2,412,267	900,471	22
	48hr	98,836,741	91,239,369	1,664,059 (1.7%)	1,356,581	3,049,400	1,142,442	27
SMRU2	6hr	90,684,985	69,739,771	8,430,032 (9.3%)	5,905,422	3,881,119	1,419,046	35
	12hr	77,712,400	66,222,208	3,883,624 (5.0%)	2,860,775	3,063,198	1,004,931	27
	18hr	67,050,680	59,482,697	2,168,040 (3.2%)	1,577,630	2,517,417	835,736	22
	24hr	79,375,619	71,671,194	1,671,182 (2.1%)	1,282,641	3,485,949	753,805	31
	30hr	75,194,646	67,547,137	1,301,870 (1.7%)	1,072,233	3,502,413	1,371,614	31
	36hr	72,467,504	65,599,562	1,324,061 (1.8%)	1,100,961	2,797,477	1,319,479	25
	42hr	60,708,594	54,433,207	1,426,784 (2.4%)	1,186,932	2,263,139	1,102,479	20
	48hr	85,453,382	74,199,241	2,739,015 (3.2%)	2,282,438	3,617,882	2,139,101	32
Control 1		127,471,747	109,474,021	2,743,501 (2.2%)	2,354,111	4,932,551	7,452,225	44
Control 2		133,823,529	116,387,796	2,754,429 (2.1%)	2,408,314	5,078,427	6,641,024	45
Control 3		122,462,033	106,772,440	2,750,379 (2.2%)	2,392,484	4,806,653	5,057,224	43
Control 4		108,387,207	92,160,848	2,471,623 (2.3%)	2,060,334	4,304,933	6,943,941	38

a. Average coverage was estimated by the total number of sequenced nucleotides divided by the total length of chromosomes.

Supplementary Table S2. Differentially expressed genes between isolates

Gene	Description
PVX_096273	DEAD/DEAH box helicase putative
PVX_085010	exodeoxyribonuclease III putative
PVX_090050	Got1 domain containing protein
PVX_003930	hypothetical protein
PVX_099835	hypothetical protein
PVX_003710	hypothetical protein
PVX_092870	hypothetical protein
PVX_089780	hypothetical protein
PVX_096030	hypothetical protein
PVX_102130	hypothetical protein
PVX_035690	hypothetical protein
PVX_123675	hypothetical protein conserved
PVX_080165	hypothetical protein conserved
PVX_122825	hypothetical protein conserved
PVX_114155	hypothetical protein conserved
PVX_113620	hypothetical protein conserved
PVX_087130	hypothetical protein conserved
PVX_123550	hypothetical protein conserved
PVX_123565	hypothetical protein conserved
PVX_087695	hypothetical protein conserved
PVX_119570	hypothetical protein conserved
PVX_087055	mRNA processing protein putative
PVX_084770	NAD(P)H-dependent glutamate synthase putative
PVX_002520	Pv-fam-b protein
PVX_091840	pyruvate dehydrogenase E1 component alpha subunit putative
PVX_101510	tryptophan-rich antigen (Pv-fam-a)
PVX_083590	variable surface protein Vir12 putative
PVX_133260	variable surface protein Vir12 putative truncated
PVX_158260	variable surface protein Vir12 truncated putative
PVX_170270	variable surface protein Vir12 truncated putative
PVX_075695	variable surface protein Vir12%2F22%2F24-related
PVX_013620	variable surface protein Vir12-like
PVX_054190	variable surface protein Vir14-related truncated
PVX_015135	variable surface protein Vir17 truncated putative
PVX_101625	variable surface protein Vir18-like
PVX_007585	variable surface protein Vir28 putative
PVX_005580	variable surface protein Vir4 putative
PVX_014630	variable surface protein Vir6 putative
PVX_088775	VIR protein
PVX_005057	VIR protein
PVX_119205	VIR protein
PVX_101630	VIR protein
PVX_090320	VIR protein pseudogene

Supplementary Table S3. List of 25 *vir* genes highly expressed in both SMRU1 and SMRU2

Gene id	Peak FPKM		Chromosome	Start	End	Strand	Product_description	Subfamily*
	SMRU1	SMRU2						
PVX_107745	6.399	5.927	AAKM01000033	10305	11525	-	variable surface protein Vir 12-like	E
PVX_108770	6.847	7.316	AAKM01000040	10972	12789	+	variable surface protein Vir 14 putative	C
PVX_120340	5.801	5.678	AAKM01000049	9527	11145	+	variable surface protein Vir12-related	E
PVX_008085	7.962	7.959	AAKM01000078	3801	5606	-	variable surface protein Vir 14-related	C
PVX_022185	6.299	7.791	AAKM01000088	3990	5971	-	variable surface protein Vir12-related	E
PVX_078195	6.017	5.287	AAKM01000217	1264	3234	+	variable surface protein Vir12-related	E
PVX_076195	5.191	5.291	AAKM01000278	1412	3053	-	variable surface protein Vir12-related	E
PVX_096965	7.163	7.456	Pv_Sal1_chr02	65458	66925	-	VIR protein	
PVX_096970	8.600	8.770	Pv_Sal1_chr02	70057	71906	-	variable surface protein Vir8-related	
PVX_096975	8.956	8.249	Pv_Sal1_chr02	74773	76790	-	VIR protein	
PVX_096980	9.304	9.067	Pv_Sal1_chr02	81156	82769	-	variable surface protein Vir putative	
PVX_096985	6.703	7.359	Pv_Sal1_chr02	87416	88742	-	variable surface proein Vir putative	
PVX_096987	8.721	9.125	Pv_Sal1_chr02	92896	93879	-	VIR protein	
PVX_096005	5.937	5.544	Pv_Sal1_chr03	884998	886408	+	variable surface protein Vir15-related	J
PVX_095990	7.163	6.254	Pv_Sal1_chr03	923820	925044	-	VIR protein	
PVX_002485	6.823	6.569	Pv_Sal1_chr04	23506	25479	-	variable surface protein Vir12-related	E
PVX_088795	5.758	5.609	Pv_Sal1_chr05	17187	18808	-	VIR protein	
PVX_090305	7.608	7.540	Pv_Sal1_chr05	1324557	1326218	+	variable surface protein Vir12-related	E
PVX_110822	5.907	6.194	Pv_Sal1_chr06	966008	967576	+	VIR protein	
PVX_004539	8.766	8.236	Pv_Sal1_chr06	987617	989020	+	variable surface protein Vir12-related	
PVX_004537	5.289	5.719	Pv_Sal1_chr06	992962	994931	+	VIR protein	E
PVX_004535	7.813	7.615	Pv_Sal1_chr06	1005422	1007102	+	variable surface protein Vir putative	C
PVX_004525	6.311	6.509	Pv_Sal1_chr06	1012546	1013849	-	variable surface protein Vir16/32-related	C
PVX_094245	5.847	6.182	Pv_Sal1_chr08	33264	35574	-	variable surface protein Vir12-like	E
PVX_113230	8.043	8.382	Pv_Sal1_chr11	2005940	2007559	+	variable surface protein Vir14-related	C

* The sub family of *vir* genes were defined by Lopez et al.[38]

Supplementary Table S4. UTR size of selected species from RefSeq database

Species	UTR5 (Median)	UTR3 (Median)
Homo sapiens (Human)	239	886
Mus musculus (Mouse)	220	821
Gallus gallus(Chicken)	89	587
Danio rerio (Zebrafish)	173	445
Drosophila melanogaster (Fly)	140	190

Supplementary Table S5. Genes with TSS selection confirmed by *de novo* transcript isoforms

Gene id	Chromosome	Strand	CDS_start	CDS_end	TSS1	TSS2	TSS3
PVX_002950	Pv_Sal1_chr04	-	407722	408342	411050	410093	408550
PVX_082845	Pv_Sal1_chr12	-	645864	647450	647792	647596	
PVX_085270	Pv_Sal1_chr13	-	1059503	1060585	1060869	1060604	
PVX_092605	Pv_Sal1_chr09	-	1563816	1565328	1565888	1566700	
PVX_113675	Pv_Sal1_chr11	-	1589156	1591168	1592995	1591821	
PVX_084310	Pv_Sal1_chr13	-	175398	177563	177983	178285	
PVX_118430	Pv_Sal1_chr12	-	2705686	2706931	2707373	2706949	
PVX_087965	Pv_Sal1_chr01	-	319741	320385	320724	321188	
PVX_094660	Pv_Sal1_chr08	-	437259	438258	438583	439065	
PVX_084670	Pv_Sal1_chr13	-	525676	526473	526508	527486	
PVX_111210	Pv_Sal1_chr06	-	641704	645240	646428	645432	
PVX_114685	Pv_Sal1_chr11	-	714007	715299	715354	716460	
PVX_122720	Pv_Sal1_chr14	-	811474	812043	813485	812608	
PVX_123260	Pv_Sal1_chr14	+	1281486	1284883	1281468	1279139	
PVX_079770	Pv_Sal1_chr10	+	82907	84242	81570	82673	
PVX_114180	Pv_Sal1_chr11	+	1194237	1195145	1194110	1193538	
PVX_086975	Pv_Sal1_chr07	+	1318150	1319927	1318072	1316640	
PVX_118255	Pv_Sal1_chr12	+	2533204	2534679	2532362	2531398	
PVX_099117	Pv_Sal1_chr07	+	520430	521014	520129	519490	
PVX_094810	Pv_Sal1_chr08	+	539072	542029	537534	538821	
PVX_081815	Pv_Sal1_chr02	+	695148	696010	694211	693437	
PVX_122710	Pv_Sal1_chr14	+	805589	806554	805259	805568	
PVX_123105	Pv_Sal1_chr14	+	1133640	1137090	1132360	1133618	
PVX_094303	Pv_Sal1_chr08	+	120982	122559	119178	119989	
PVX_098685	Pv_Sal1_chr07	+	126033	127870	125368	125722	
PVX_092540	Pv_Sal1_chr09	+	1472476	1475094	1470326	1471771	
PVX_123635	Pv_Sal1_chr14	+	1609822	1610505	1608466	1609357	
PVX_118162	Pv_Sal1_chr12	+	2454218	2454979	2453764	2453938	
PVX_091095	Pv_Sal1_chr09	+	261138	262145	261090	260433	
PVX_083195	Pv_Sal1_chr12	+	330941	332695	330322	329966	
PVX_080215	Pv_Sal1_chr10	+	485026	486172	484482	484886	
PVX_096271	Pv_Sal1_chr03	+	650899	652743	650366	650377	
